# Glutamatergic Neurons in the Preoptic Hypothalamus Promote Wakefulness, Destabilize NREM Sleep, Suppress REM Sleep, and Regulate Cortical Dynamics

**DOI:** 10.1523/JNEUROSCI.2718-20.2021

**Published:** 2021-04-14

**Authors:** Alejandra Mondino, Viviane S. Hambrecht-Wiedbusch, Duan Li, A. Kane York, Dinesh Pal, Joaquin González, Pablo Torterolo, George A. Mashour, Giancarlo Vanini

**Affiliations:** ^1^Department of Anesthesiology, University of Michigan, Ann Arbor, Michigan 48109-5615; ^2^Center for Consciousness Science, University of Michigan, Ann Arbor, Michigan 48109; ^3^Neuroscience Graduate Program, University of Michigan, Ann Arbor, Michigan 48109-2215; ^4^Departamento de Fisiología, Facultad de Medicina, Universidad de la República, Montevideo, 11200, Uruguay

**Keywords:** arousal, consciousness, DREADDs, gamma, sleep fragmentation, slow oscillations

## Abstract

Clinical and experimental data from the last nine decades indicate that the preoptic area of the hypothalamus is a critical node in a brain network that controls sleep onset and homeostasis. By contrast, we recently reported that a group of glutamatergic neurons in the lateral and medial preoptic area increases wakefulness, challenging the long-standing notion in sleep neurobiology that the preoptic area is exclusively somnogenic. However, the precise role of these subcortical neurons in the control of behavioral state transitions and cortical dynamics remains unknown. Therefore, in this study, we used conditional expression of excitatory hM3Dq receptors in these preoptic glutamatergic (Vglut2^+^) neurons and show that their activation initiates wakefulness, decreases non-rapid eye movement (NREM) sleep, and causes a persistent suppression of rapid eye movement (REM) sleep. We also demonstrate, for the first time, that activation of these preoptic glutamatergic neurons causes a high degree of NREM sleep fragmentation, promotes state instability with frequent arousals from sleep, decreases body temperature, and shifts cortical dynamics (including oscillations, connectivity, and complexity) to a more wake-like state. We conclude that a subset of preoptic glutamatergic neurons can initiate, but not maintain, arousals from sleep, and their inactivation may be required for NREM stability and REM sleep generation. Further, these data provide novel empirical evidence supporting the hypothesis that the preoptic area causally contributes to the regulation of both sleep and wakefulness.

**SIGNIFICANCE STATEMENT** Historically, the preoptic area of the hypothalamus has been considered a key site for sleep generation. However, emerging modeling and empirical data suggest that this region might play a dual role in sleep-wake control. We demonstrate that chemogenetic stimulation of preoptic glutamatergic neurons produces brief arousals that fragment sleep, persistently suppresses REM sleep, causes hypothermia, and shifts EEG patterns toward a “lighter” NREM sleep state. We propose that preoptic glutamatergic neurons can initiate, but not maintain, arousal from sleep and gate REM sleep generation, possibly to block REM-like intrusions during NREM-to-wake transitions. In contrast to the long-standing notion in sleep neurobiology that the preoptic area is exclusively somnogenic, we provide further evidence that preoptic neurons also generate wakefulness.

## Abstract (precis)

Clinical and experimental data from the last nine decades indicate that the preoptic area of the hypothalamus is a critical node in a brain network that controls sleep onset and homeostasis. By contrast, we recently reported that a group of glutamatergic neurons in the lateral and medial preoptic area increases wakefulness, challenging the long-standing notion in sleep neurobiology that the preoptic area is exclusively somnogenic.

## Introduction

Since the beginning of the last century, the preoptic area of the hypothalamus has been considered a critical site for sleep generation. In the early 1900s, von Economo's work revealed that patients suffering severe insomnia after encephalitis lethargica had extensive lesions within the rostral hypothalamus (von Economo, 1930). Consistent with this observation, subsequent studies demonstrated that lesioning of the preoptic area causes a prolonged, severe insomnia in cats (Sallanon et al., 1989) and rats (Nauta, 1946; John et al., 1994; Lu et al., 2000; Eikermann et al., 2011), and transplantation of fetal preoptic cells into the preoptic area partially restored sleep quantity in previously lesioned insomniac rats (John et al., 1998). Infusion of adenosine (or adenosine analogs) into the preoptic region increases sleep and decreases wakefulness (Ticho and Radulovacki, 1991; Mendelson, 2000), whereas pharmacologic inhibition of this region reduces sleep (Lin et al., 1989; M. N. Alam and Mallick, 1990; Benedetto et al., 2012). Additionally, evidence from single-cell recording and cFos studies confirmed that the median preoptic nucleus (MnPO) as well as ventrolateral and medial preoptic area contain neurons that are mainly active during non-rapid eye movement (NREM) sleep (Koyama and Hayaishi, 1994; Szymusiak et al., 1998; Suntsova et al., 2002; Takahashi et al., 2009; Sakai, 2011; M. A. Alam et al., 2014; Zhang et al., 2015; Chung et al., 2017; Harding et al., 2018) and, to a lesser extent, during rapid eye movement (REM) sleep (Koyama and Hayaishi, 1994; Lu et al., 2002; Suntsova et al., 2002; Gvilia et al., 2006; Dentico et al., 2009; Sakai, 2011; M. A. Alam et al., 2014). Importantly, a subset of these cells increases its activity during periods of prolonged wakefulness with increased sleep pressure, suggesting a role in the regulation of both NREM and REM sleep homeostasis (Gvilia et al., 2006; Dentico et al., 2009; Todd et al., 2010; M. A. Alam et al., 2014; Gvilia et al., 2017).

Several lines of evidence show that a group of GABAergic neurons (some of which coexpress galanin) distributed within the median, medial-lateral, and ventrolateral preoptic area are sleep-active and innervate monoaminergic arousal-promoting systems (Sherin et al., 1998; Gaus et al., 2002; Uschakov et al., 2006; Hsieh et al., 2011; Chung et al., 2017). Activation of preoptic GABAergic and galaninergic neurons promotes NREM sleep (Chung et al., 2017; Harding et al., 2018; Kroeger et al., 2018; Vanini et al., 2020) and lesion (Lu et al., 2000; Ma et al., 2019), or neuromodulation of these neurons (Harding et al., 2018; Kroeger et al., 2018) alters the EEG during sleep-wake states. Furthermore, a recent study using activity-dependent tagging and subsequent stimulation of previously tagged neurons showed that a subgroup of glutamatergic neurons within the median and medial preoptic nucleus of the hypothalamus promotes body cooling and NREM sleep (Harding et al., 2018). However, contrary to the prevailing idea in the field that virtually all preoptic neurons that regulate sleep-wake states are somnogenic, we recently reported that a region including the ventrolateral preoptic nucleus (VLPO) and the ventral half of the medial and lateral preoptic area, collectively referred in the current study as medial-lateral preoptic region, contains glutamatergic neurons that promote wakefulness (Vanini et al., 2020).

Here we used a chemogenetic stimulation approach to investigate further the role of these preoptic glutamatergic neurons in the regulation of sleep-wake states as well as state transitions and cortical dynamics, which have not yet been examined. We show that stimulation of glutamatergic neurons in the medial-lateral preoptic region causes a transient increase in wakefulness and a decrease in both NREM and REM sleep. Activation of these neurons also produces a high degree of NREM sleep fragmentation and state instability, a “lighter” NREM sleep state, and a long-lasting suppression of REM sleep. Thus, our data suggest that a subset of preoptic glutamatergic neurons may initiate, but not maintain, arousal from sleep, and their inactivation might be required for NREM stability and REM sleep generation. Furthermore, these results provide novel empirical evidence that the preoptic area plays a dual role in the regulation of both sleep and wakefulness.

## Materials and Methods

### 

#### Mice

Vglut2-IRES-Cre (Slc17a6tm2(cre)Lowl/J; stock #016963) mice were purchased from The Jackson Laboratory, bred at the University of Michigan animal care facility, and genotyped (Transnetyx) before weaning. Adult male mice used in this study (*n* = 41) were housed in groups in a 12 h light:dark cycle (lights on at 6:00 A.M.) with free access to food (PicoLab Laboratory Rodent Diet 5LOD; LabDiet) and water. The temperature in the housing and testing rooms was maintained at 22°C. All experiments were approved by the Institutional Animal Care and Use Committee and the Institutional Biosafety Committee, and were conducted in accordance with the recommendations published in the *Guide for the care and use of laboratory animals* (Ed 8, National Academies Press, Washington, DC, 2001).

#### Viral vector and drugs

For Cre-recombinase-dependent expression of the excitatory designer receptor hM3Dq in preoptic glutamatergic (Vglut2^+^) neurons, we used the adeno-associated viral vector AAV-hSyn-DIO-hM3D(Gq)-mCherry (Addgene; catalog #50459-AAV5) (Krashes et al., 2011; Vanini et al., 2020). In a separate group of Vglut-Cre mice used for control experiments, we used the Cre-dependent vector AAV-hSyn-DIO-mCherry (Addgene; catalog #50459-AAV5) that lacked the coding sequence for the designer receptor and only contained the fluorescent reporter mCherry. The titer of the viral solutions was 3.7-7.8 × 10^12^ copies per ml. DMSO and clozapine-N-oxide (CNO, catalog #C0832-5MG; agonist at hM3Dq receptors) were purchased from Sigma Millipore. A stock solution of CNO (0.1 mg/ml in saline solution containing 0.5% DMSO) was prepared and stored in aliquots, and then frozen at −20°C until use. Before each experiment, an aliquot of the stock solution was thawed at room temperature protected from the light until injection time. Mice received 1 mg/kg CNO or a vehicle control solution (VEH, saline with 0.5% DMSO) by intraperitoneal injection; the injection volume was 0.1 ml per 10 g of body weight (Vanini et al., 2020).

#### Viral injection and electrode implantation for sleep studies

The procedure for vector injection into the preoptic area was performed as described previously (Vanini et al., 2020). Mice were anesthetized in an induction chamber with 5.0% isoflurane (Hospira) in 100% O_2_. The delivered concentration of isoflurane was monitored continuously by spectrometry (Cardiocap/5; Datex-Ohmeda). Following anesthetic induction, mice received preemptive analgesia (5 mg/kg carprofen; s.c.), and were immediately placed in a Kopf model 962 stereotaxic frame fitted with a mouse adaptor and a mouse anesthesia mask (models 922 and 907, respectively; David Kopf Instruments). Thereafter, the concentration of isoflurane was reduced and maintained at 1.6%-2.0% throughout the surgical procedure. Core body temperature was maintained at 37°C-38°C with a water-filled pad connected to a heat pump (Gaymar Industries). For the medial-lateral preoptic region, vector injections were performed bilaterally (36 nl on each side at the stock concentration provided by Addgene); stereotaxic coordinates: AP = 0.15 mm, ML = ±0.5 mm and DV = –5.0 mm from bregma (Vanini et al., 2020). Injections were performed at a rate of 5 nl/min using a Hamilton Neuros Syringe 7000 (5 ml) mounted on a microinjection syringe pump, driven by a digital Micro2T controller (model UMP3T-2; World Precision Instruments). After each injection, the syringe was maintained in position for an additional 5 min to avoid vector reflux. We previously confirmed that this volume, stereotaxic coordinates, and injection procedure yielded a reliable expression of hM3Dq receptors in the target region (Vanini et al., 2020). The scalp incision was closed with sutures, and mice were placed under a controlled heat source until full recovery. Mice were then returned to the vivarium and group-housed with their respective littermates. Analgesia was maintained with carprofen (5 mg/kg every 24 h) for a minimum of 48 h after surgery.

Three weeks following vector injection, mice were anesthetized with isoflurane and EEG electrodes (8IE3632016XE, E363/20/1.6/SPC; Plastics One) were implanted above the right frontal (AP = 1.5 mm, ML = 2.0 mm from bregma) and occipital (AP = –3.2 mm, ML = 3.0 mm from bregma) cortices. A reference screw electrode was implanted over the cerebellum, and a pair of EMG electrodes (8IE36376XXXE, E363/76/SPC; Plastics One) was inserted bilaterally into the dorsal neck muscles. All electrode pins were then inserted into an electrode pedestal (MS363; Plastics One) that was affixed to the skull with dental cement (Fast Cure Powder/Liquid, Product #335201; GC America). Thereafter, the delivery of isoflurane was discontinued, and mice were allowed to recover following the same postoperative care protocol outlined above. Mice were allowed a minimum of 10 d before experiments began.

#### EEG/EMG data acquisition

At least 1 week after the surgery for implantation of EEG/EMG electrodes, mice were conditioned to handling, simulating drug injection and tethering in the recording setup for 5 d. On the day of the experiment, mice received an injection of VEH or CNO and EEG/EMG signals were recorded for 6 h. The injection and recording start time was 10:00 A.M. (ZT04), when homeostatic sleep pressure is high, an optimal time to study the wakefulness-promoting effect of the targeted neurons. Monopolar EEG and bipolar EMG signals were amplified (×1000) and digitized (sampling rate = 1024 Hz), respectively, with a model 1700 AC amplifier (A-M Systems) and a Micro3 1401 acquisition unit and Spike2 software (Cambridge Electronic Design); notch filters were used for each mouse recording (both after VEH and CNO) when 60 Hz electrical noise was present. All signals were bandpass filtered between 0.1 and 500 Hz (EEG) and between 10 and 500 Hz (EMG). Mouse behaviors were video recorded, in synchrony with the electrophysiologic recordings in Spike2.

#### Analysis of sleep-wake states

States of wakefulness, NREM sleep, and REM sleep were manually scored in Spike2 in 5 s epochs using standard criteria described previously (Vanini et al., 2020). Wakefulness was defined by low-amplitude, high-frequency EEG activity accompanied by an active EMG characterized by high tone with phasic movements. NREM sleep was recognized by high-amplitude, low-frequency EEG waveforms along with a low muscle tone. REM sleep was identified by low-amplitude, high-frequency EEG signals with prominent, regular theta rhythm (particularly evident in the occipital cortex), and EMG atonia. Total time spent in wakefulness, NREM sleep, and REM sleep, the average duration and number of bouts for each vigilance state, was analyzed over the 6 h recording period, as well as in 1 h bins to assess the mean duration of the treatment effect. The mean latency to NREM sleep and REM sleep onset was compared between treatment conditions. Additionally, we reanalyzed sleep recordings from a group of Vglut2-Cre mice used in a recent study with confirmed hM3Dq receptor expression within the MnPO of the hypothalamus (Vanini et al., 2020). The reanalysis extended the assessment of sleep recordings from 3 to 6 h and was performed by the same investigator using the same scoring criteria as in the study by Vanini et al. (2020). Furthermore, the viral vector, surgical procedures, experimental design, and dose of CNO were identical in both studies. These mice served in the current study as a site-control group.

#### Sleep fragmentation analysis

An initial exploratory analysis revealed that chemogenetic activation of preoptic glutamatergic neurons substantially increased the number and decreased the duration of wakefulness and NREM sleep bouts, revealing a robust sleep fragmentation. Therefore, to further characterize this phenomenon, we calculated the stability of wake and NREM sleep states, between- and within-state transition probabilities, as well as a fragmentation index (FI). First, the mean number of transitions from NREM to wakefulness was quantified over the 6 h recording period and for every 1 h bin. The stability of each state was calculated using a Markov memoryless model. This is a regression-like method in which variables are modeled as a function of the previous observations (J. W. Kim et al., 2009; Perez-Atencio et al., 2018). In this formalism, the probability of being in one state is determined by the previous state and the transition probability from one state to another. The probability of each specific state was calculated by the number of times that this state was scored over the total number of epochs recorded and the transition probability was obtained as the conditional probability P(X/Y), that is, the number of times that State Y transitioned to State X in the following epoch, divided by the number of epochs of the Y state. Next, we calculated an FI for wakefulness and NREM sleep (codes are available at https://github.com/joaqgonzar/Fragmentation_Index). We defined FI as FI = 1 – p(X/X), that is, 1 minus the probability of transitioning from the State X to the same State X. This implies that FI = 1 only if the state is completely fragmented, in other words, if the probability of transitioning to the same state equals 0. Finally, as the sleep-wake cycle is characterized by bouts of short and long duration, we generated histograms of bout length for wakefulness and NREM sleep. These plots were obtained by counting the number of events that occurred in each increment of 10 s for wakefulness and 50 s for NREM sleep.

#### Determination of changes in body temperature after activation of glutamatergic neurons in the medial-lateral preoptic region

The effect of the activation of medial-lateral preoptic glutamatergic neurons on core body temperature was evaluated in two different experiments using a separate group of mice. The first set of experiments tested whether stimulation of these neurons alters body temperature. Mice were briefly and gently restrained for 10-15 s each time, and core body temperature was measured using a lubricated mouse rectal probe (model 600-1000, Barnant). After obtaining a stable temperature reading, the probe was removed and the animal was returned to its cage until the next measurement. Temperature measurements were obtained before the injection of VEH or CNO (1.0 mg/kg) and in 10 min intervals for 90 min after injection. If a temperature drop was detected, mice were placed under a heating lamp in their respective home cages between testing intervals. The second set of experiments was designed to evaluate the duration of temperature changes after CNO administration, relative to the duration of the sleep studies. Using the same procedure described above, temperature measurements were obtained in 30 min intervals for 6 h after CNO injection.

#### EEG signal processing and analysis

EEG analysis (power, coherence, connectivity, and complexity; 0.1-115 Hz) was conducted on artifact-free, nontransition epochs during the initial 3 h block after VEH and CNO injection. Digitized, raw EEG signals in frontal and occipital channels were exported from Spike2 software into MATLAB (version 2019b; The MathWorks) and downsampled to 512 Hz (resample.m function in MATLAB signal processing toolbox). Because notch filters were applied to EEG channels in some recordings (always in VEH/CNO pairs, for consistency), frequencies between 45 and 75 Hz were not considered for the analysis. The power-line interference (60 and 120 Hz), if present, was removed using multitaper regression technique and Thomas *F* statistics implemented in Cleanline plugin for EEGLAB toolbox (Delorme and Makeig, 2004). The 3-h-long recording blocks were segmented into 5 s windows with no overlap. All EEG measures were calculated from *n* = 10 mice (#541 was excluded because of poor signal quality that precluded its use in these analyses) in each window and then averaged over all available windows to obtain the mean values for each vigilance state, treatment condition, and mouse.

##### Spectral power and coherence analysis

The EEG power spectrum was estimated using the multitaper method in Chronux analysis toolbox (Mitra and Bokil, 2008) with a window length = 5 s without overlap, time-bandwidth product = 2, and number of tapers = 3. For each mouse in each treatment group, the mean power spectrum in each behavioral state (wake, NREM, REM) was obtained by averaging the power spectra across all available windows in frontal and occipital channels, respectively. Furthermore, the power values were calculated for slow oscillations (0.5-1 Hz) as well as δ (1-4 Hz), theta (4-9 Hz), σ (9-15 Hz), β (15-30 Hz), low γ (30-45 Hz), and high γ (75-115 Hz) frequency bands. Power values were normalized for each mouse as the mean power of each frequency band divided by the sum of the power in all bands (i.e., total power).

Cortical coherence (a measure of undirected functional connectivity) between frontal and occipital channels was quantified by the multitaper time-frequency coherence method in Chronux analysis toolbox (Mitra and Bokil, 2008), with the same parameters described above for the spectral power analysis. The frontal-occipital coherence as a function of frequency was thus obtained by averaging the coherence over time in each behavioral state, for each mouse and treatment group. Coherence values were normalized by the Fisher's *z*-transform (Miranda de Sá et al., 2009). The same frequency bands used in the spectral power analysis were assessed in the coherence analysis.

##### Analysis of directed connectivity

We used normalized symbolic transfer entropy (NSTE) to assess directed connectivity between frontal and occipital cortices. NSTE is an information theoretic measure, and our previous studies have validated its use to measure cortical connectivity changes in humans (Lee et al., 2013) and rats (Borjigin et al., 2013; Pal et al., 2016, 2020). In the calculation of NSTE, three parameters are required: embedding dimension ($d_{E}$), time delay (τ), and transfer time (δ). We filtered the frontal and occipital EEG signals into the frequency bands as described above and segmented the filtered data into nonoverlapped 5 s windows. We followed the methods used in previous studies (D. Li et al., 2017; Pal et al., 2020) and fixed the embedding dimension $d_{E}$ = 3; as the time delay τ defines a broad frequency-specific window of sensitivity for NSTE (Jordan et al., 2013; Sitt et al., 2014; Ranft et al., 2016), we used τ = 64, 28, 17, 9, 6, 2, corresponding to δ (0.5-4 Hz), theta, σ, β, low- and high-γ, respectively. For each window, we searched the transfer time δ = 1-50 (corresponding to ∼2-100 ms) and selected the one that generated maximum feedback (from frontal to occipital) and feedforward (from occipital to frontal) NSTE, respectively. For statistical comparisons, the averaged connectivity values were calculated over the studied behavioral states for each mouse and treatment group.

##### Complexity analysis

We used Lempel-Ziv complexity (LZc) to quantify the dynamic changes in EEG signals from frontal and occipital cortices in the different behavioral states between the two treatment conditions. LZc is a method of symbolic sequence analysis (Lempel and Ziv, 1976) that has been shown to be a valuable tool to investigate the temporal or spatiotemporal complexity of brain activity (Schartner et al., 2017; D. Li and Mashour, 2019; Brito et al., 2020; Pal et al., 2020). Given the limited number of EEG channels in this study, we assessed only temporal complexity in frontal and occipital channels; spatiotemporal complexity was not evaluated. EEG signals were detrended using local linear regression (locdetrend function in Chronux analysis software) and a lowpass filter was applied at 115 Hz (but excluding the frequencies at 45-75Hz) via Butterworth filter of order 4 (butter and filtfilt functions in MATLAB signal processing toolbox). The Hilbert transform of the signal was used to calculate the instantaneous amplitude, which was then segmented into 5 s windows without overlapping and binarized using its mean value as the threshold for each channel (Schartner et al., 2017). LZc analysis estimates the complexity of a finite series of numbers by computing the number of times that a different subsequence of consecutive characters, or “word,” is found within that series. To assess the signal complexity beyond the spectral changes, we generated surrogate data through phase randomization while preserving the spectral profiles of the signal (Schartner et al., 2015, 2017; Brito et al., 2020; Pal et al., 2020), and normalized the original LZc by the mean of the LZc values from *N* = 50 surrogate time series for each recording. The resultant LZc values were then averaged across all the windows as the estimate of the complexity for wakefulness, NREM sleep, and REM sleep. Higher values of LZc reflect higher complexity of the EEG signal. Analysis of spectral power, connectivity, and complexity during REM sleep was precluded by the profound suppression of REM sleep in the CNO group, which led to an insufficient sample size.

#### Immunohistochemistry and histologic verification of designer receptor expression; confirmation of neuronal activation after CNO administration

After the last sleep experiment, mice were deeply anesthetized with isoflurane and perfused transcardially with ice-cold 0.1 m PBS, pH 7.4, followed by 5% formalin for 10 min using a MasterFlex perfusion pump (Cole Palmer). A subset of mice previously injected with AAV-hM3Dq-mCherry (not used for sleep or temperature experiments) was perfused 90 min after an injection of VEH or CNO (*n* = 4 mice in each treatment group) for analysis of cFos expression in glutamatergic neurons of the medial-lateral preoptic region; the time of perfusion was 2:00 P.M. (ZT08) to minimize spontaneous cFos expression in the preoptic area. After perfusion, the brains were removed and postfixed in 5% formalin overnight at 4°C. Subsequently, brains were cryoprotected with 20% sucrose in PBS for 1-2 d, and then transferred to 30% sucrose for an additional 2-3 d. Thereafter, brains were frozen in Tissue–Plus (Fisher Healthcare) and sectioned coronally at 40 µm using a cryostat (CM1950, Leica Microsystems). Brain sections that contained the VLPO were collected and blocked in PBS containing 0.25% Triton X-100 and 3% normal donkey serum (Vector Laboratories) for 60 min at room temperature. Thereafter, sections were immunolabeled for mCherry, or sequentially processed for double immunohistochemistry for mCherry and cFos. First, we incubated the tissues overnight at room temperature in primary antiserum (rat monoclonal anti-mCherry 1:30,000; Thermo Fisher Scientific, catalog #M11217). For cFos detection, sections were incubated in a rabbit polyclonal anti-cFos (1:5000; Sigma Millipore, catalog #ABE457) overnight at room temperature. Next, sections were washed in PBS and incubated for 2 h at room temperature in a donkey anti-rat secondary antiserum (1:500; AlexaFluor-594, Thermo Fisher Scientific, catalog #A-21209 for mCherry and 1:500; AlexaFluor-488, Thermo Fisher Scientific, catalog #A-21206 for cFos). Last, brain sections were washed with PBS, float-mounted on glass slides, and coverslipped with SlowFade Diamond (S36972 or S36973; Invitrogen). Brain sections containing the target region were then examined by means of fluorescence microscopy (BX43, Olympus America). The brain regions in which the designer receptor hM3Dq were expressed were identified with the aid of a mouse brain atlas (Paxinos and Franklin, 2001). For stimulation experiments, only those mice that had reliable expression of designer receptors within the VLPO and adjacent areas were included in the analysis. The number mCherry-positive neurons that expressed cFos was visually examined by an experienced investigator and was quantified as the percentage of total neurons expressing mCherry in the medial-lateral preoptic region. Vglut2-Cre mice that received an AAV-hSyn-DIO-hM3D(Gq)-mCherry injection but did not express designer receptors were used as controls in the sleep studies described above.

#### Statistical analyses

Statistical comparisons were performed with PRISM version 7.0 (GraphPad Software). All data were tested for normality and are presented as mean ± SEM; *t* test and ANOVA results are reported as “*t*(DF) = …” and “*F*(DFn, DFd) = …,” respectively. A *p* < 0.05 was considered statistically significant. The effect of stimulation of preoptic glutamatergic neurons on total state duration, number of bouts, bout duration, and number of NREM-to-W transitions over the 6 h recording period was assessed by a one-tailed paired *t* test (Bonferroni correction was used for analysis of the data reported in Figs. 2*A-C*, 7*A-C*, and 8*A-C*). The directional hypothesis that predicted the nature of the effects of chemogenetic stimulation of preoptic glutamatergic neurons on these sleep-wake parameters was based on previously published data (Vanini et al., 2020). Differences in sleep-wake parameters for each 1 h block were evaluated by a two-way repeated-measures ANOVA followed by a Bonferroni multiple-comparisons test. The effect of CNO injection on the latency to NREM sleep and REM sleep onset was analyzed by a one-tailed paired *t* test and by survival analysis. The effect of CNO administration on core body temperature was assessed by a two-way ANOVA followed by Dunnett (within-group comparisons) and Tukey (between-group comparisons) tests. The treatment effect on EEG spectral power, Z'coherence, and NSTE (connectivity measures) for each frequency band was also assessed by a two-way repeated-measures ANOVA followed by a Bonferroni multiple-comparisons test. Changes in LZc were analyzed by a two-tailed paired *t* test. The effect of CNO administration on the FI over the 6 h recording period was determined by a Wilcoxon signed-rank test; the Benjamini-Hochberg correction for multiple-comparisons with a false discovery rate of 0.05 was used for the hour-by-hour analysis. Differences between treatment conditions in the distribution histograms were compared by the Kolmogorov-Smirnov test. Last, the relationship between REM sleep time (averaged for each mouse across the 6 h recording period) and the FI or the number of NREM-wake transitions was estimated using the Spearman correlation coefficient.

## Results

### Anatomical localization of designer receptors and confirmation of neuronal activation by designer receptors

**Figure 1.**
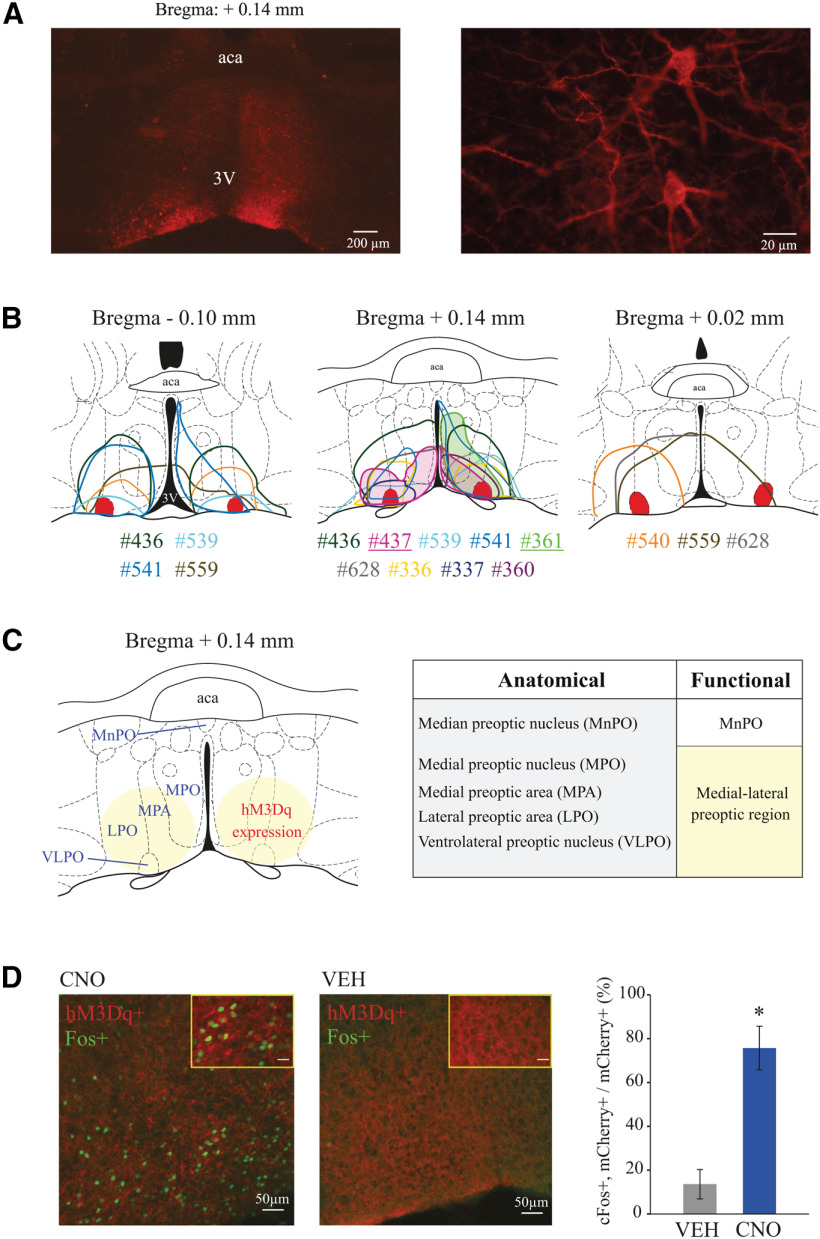
Histologic confirmation of hM3Dq receptor expression in the preoptic area of Vglut2-Cre mice used in sleep studies, and confirmation of neuronal activation caused by CNO administration. ***A***, Left, Right, Examples of low- and high-magnification photographs, respectively, of mCherry immunohistochemical staining (red), indicating the expression of the excitatory designer receptor hM3Dq within the VLPO of a Vglut2-Cre mouse. ***B***, Color-coded depiction of the area of hM3Dq receptor expression is represented on coronal schematics of the preoptic area modified from a mouse brain atlas (Paxinos and Franklin, 2001). Middle, Filled areas of hM3Dq expression highlight mice #437 and #361 (also indicated by underlined numbers below the brain schematic), which had the largest increase in sleep and wakefulness, respectively. For anatomic reference, solid red represents the bilateral sites corresponding to the VLPO. Color-matched identification numbers for each mouse used in the sleep studies (*n* = 11) are listed below each panel. ***C***, Schematic of the preoptic area illustrating relevant anatomic subdivisions and main area of designer receptor expression (yellow circles). Right, Chart compares the anatomic subdivisions and functional nomenclature used in this study. Based on the uniform response to chemogenetic stimulation, different from that observed in MnPO studies (Vanini et al., 2020), the area of hM3Dq receptor expression in the current study is referred to as the medial-lateral preoptic region. ***D***, cFos expression (green nuclei) in mCherry-positive (red) neurons in the medial-lateral preoptic region after CNO (1.0 mg/kg; *n* = 4 mice) and VEH (*n* = 4) administration. Graph plots the percentage (mean ± SEM) of mCherry-positive neurons that also expressed cFos over the total count of mCherry-positive neurons. Two-tailed unpaired *t* test was used for statistical comparison between treatment conditions. *Significant difference (*p* < 0.05) relative to control. Scale bars: high-magnification photographs (insets), 20 µm. aca, Anterior commissure; 3V, third ventricle.

As described in our previous publication (Vanini et al., 2020), histologic examination of vector injection sites revealed that neurons expressing hM3Dq receptors were localized within the VLPO and the adjacent region encompassing (from medial to lateral) the ventral aspect of the medial preoptic nucleus as well as medial and lateral preoptic area (Fig. 1). As stated above, in the current study, we refer to this region collectively as medial-lateral preoptic. There was no correlation between the area of receptor expression within the preoptic region and the stimulation effects on sleep-wake variables. Importantly, there was no receptor expression in glutamatergic neurons, nor in fibers, within any structure of the basal forebrain region (i.e., horizontal limb of the diagonal band of Broca and substantia innominata), just lateral to the preoptic region of the hypothalamus. Analysis of cFos expression confirmed that, relative to control, CNO administration activated glutamatergic neurons in the medial-lateral preoptic region (two-tailed *t* test, *t*_(6)_ = 5.17; *p* = 0.0021).

### Activation of medial-lateral preoptic glutamatergic neurons increased wakefulness and reduced NREM and REM sleep

**Figure 2.**
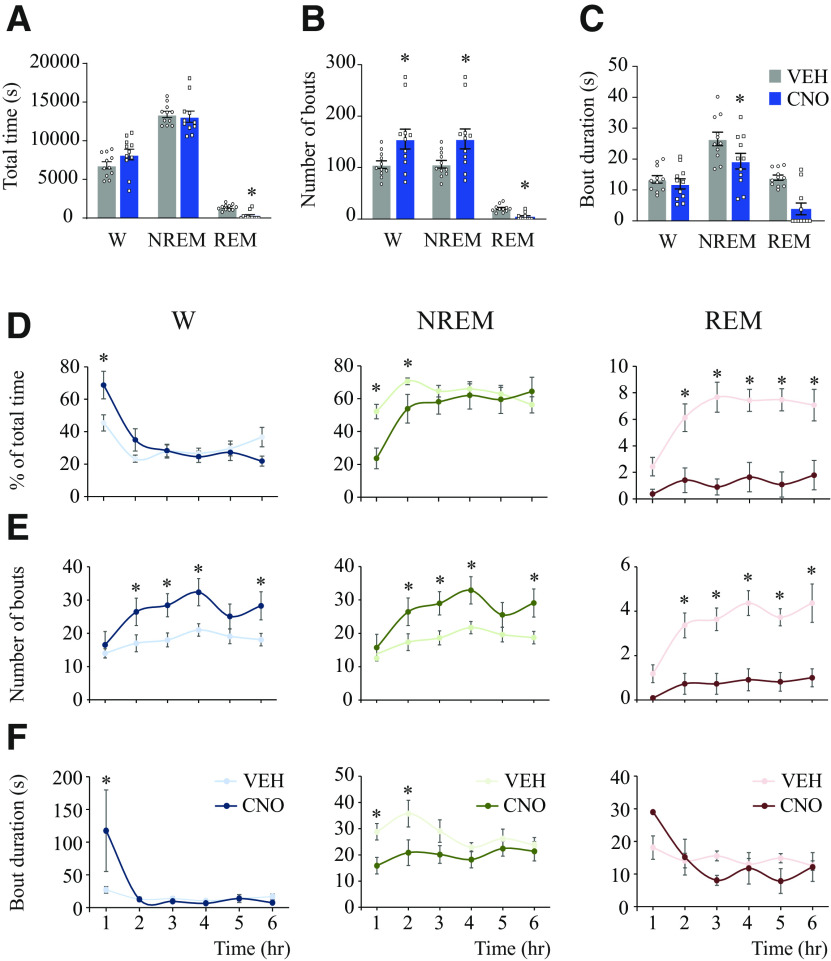
Chemogenetic activation of glutamatergic neurons in the medial-lateral preoptic region increased wakefulness and decreased NREM and REM sleep. ***A***, Time spent in wakefulness, NREM sleep, and REM sleep, averaged across the 6 h recording period, after administration of VEH and CNO (1.0 mg/kg) in *n* = 11 mice. ***B***, Effect on the number of wakefulness, NREM sleep, and REM sleep bouts. ***C***, Changes in the mean duration of wake, NREM sleep, and REM sleep bouts. Additionally, analyses of sleep-wake parameters were conducted in 1 h blocks after VEH (lighter color) and CNO (darker color) injection. ***D***, Percent of total time in wakefulness, NREM sleep, and REM sleep. ***E***, Number of bouts per state. ***F***, Mean duration of wake, NREM sleep, and REM sleep bouts. ***A–C***, A one-tailed paired *t* test with Bonferroni correction was used for statistical comparisons. ***D–F***, Two-way, repeated-measures ANOVA followed by a Sidak test adjusted for multiple comparisons was used to statistically compare sleep-wake parameters shown as a function of time and treatment condition. Data are mean ± SEM. *Significant difference (*p* < 0.05) relative to control.

**Figure 3.**
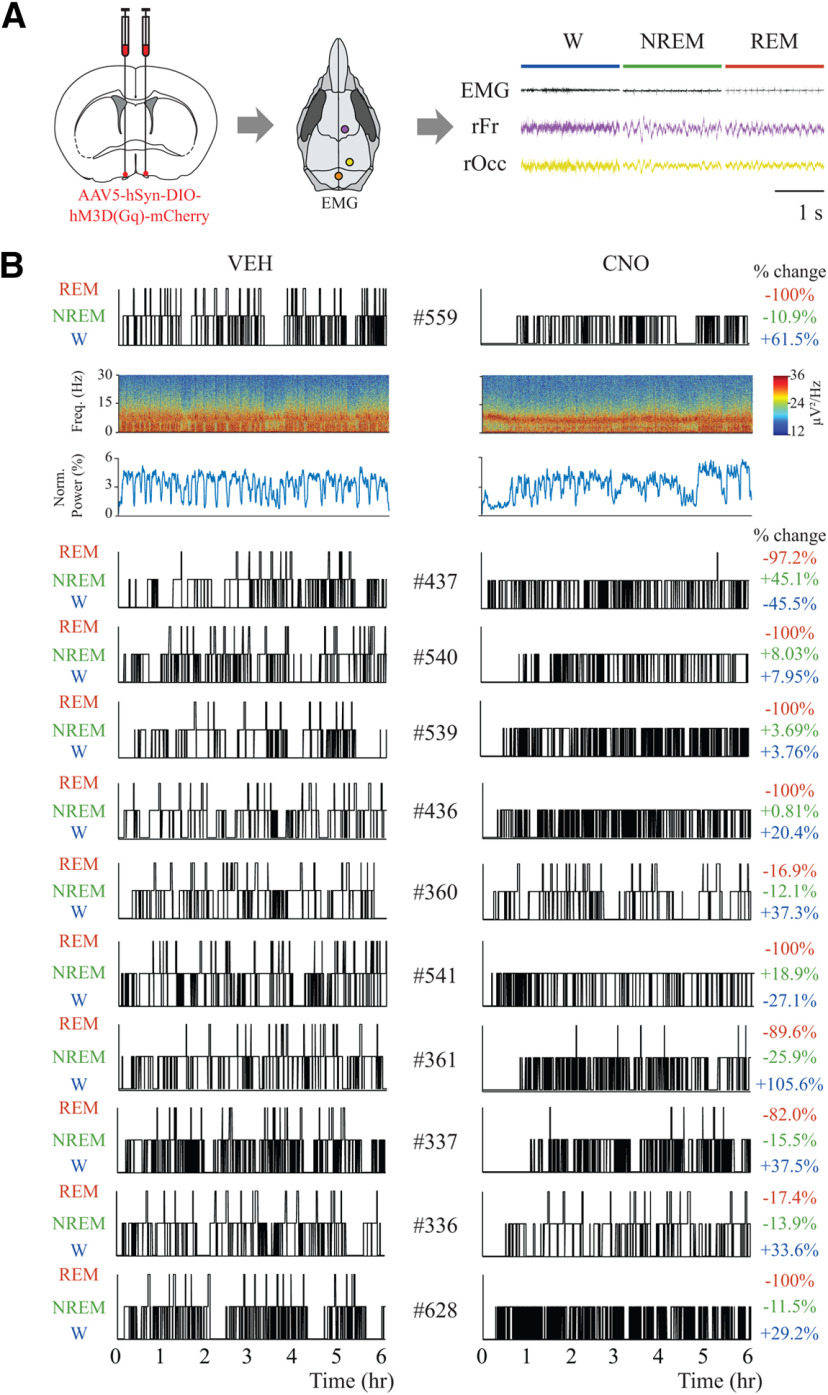
Chemogenetic activation of glutamatergic neurons in the medial-lateral preoptic region altered sleep-wake patterns. ***A***, Schematic representation of bilateral injections of a Cre-dependent adeno-associated virus for expression of the excitatory designer receptor hM3Dq into the medial-lateral preoptic region of Vglut2-Cre mice. Three weeks after the injection, mice were implanted with electrodes for recording the EEG from the right frontal (purple) and right occipital (yellow) cortex. A reference electrode was placed over the cerebellum (orange), and two electrodes were also implanted bilaterally in the neck muscles for recording the EMG. Right, Representative EEG and EMG signals from a mouse during wakefulness, NREM sleep, and REM sleep. ***B***, Hypnogram pairs illustrate the temporal organization of sleep-wake states after VEH (left panels) and 1.0 mg/kg CNO (right panels) for each mouse (*n* = 11). The height of the bars (from lowest to highest) represents the occurrence of wakefulness (W), NREM sleep, and REM sleep. Time 0 on the abscissa indicates the time at which the mouse received the injection of VEH or CNO. Mouse identification numbers are listed between each pair of hypnograms. Individual differences relative to control (% change) in the time spent in wakefulness (blue), NREM sleep (green), and REM sleep (red) after CNO injection are shown to the right of each CNO hypnogram. Representative spectrograms and power plots below the top two hypnograms corresponding to Mouse #559 show, respectively, state- and treatment-specific changes in frontal power density and normalized δ power with median filter across the 6 h recording session.

Previously published data validated the viral vector for stimulation of preoptic neurons and showed that activation of preoptic glutamatergic neurons causes a robust increase in wakefulness (Vanini et al., 2020). The present study used a chemogenetic stimulation method and thorough analysis to better understand the role of these neurons in the regulation of behavioral states, sleep-wake state transitions, and cortical dynamics. Figure 2 summarizes the effect of stimulation of preoptic glutamatergic neurons on sleep-wake variables (time spent in wakefulness, NREM sleep, and REM sleep as well as the mean number and duration of bouts for each state) averaged across the 6 h postinjection period. Compared with VEH, CNO administration significantly reduced the time spent in REM sleep (*t*_(10)_ = 6.72; *p* < 0.0001) (Fig. 2*A*); this state was completely abolished in 6 of the 11 mice studied (Fig. 3). There was no significant difference in the time spent in wakefulness (*t*_(10)_ = 0.58; *p* = 0.2870) and NREM sleep (*t*_(10)_ =1.27; *p* = 0.1166). Activation of preoptic glutamatergic neurons significantly increased the number of wakefulness and NREM sleep bouts (*t*_(10)_ = 2.58; *p* = 0.0136 and *t*_(10)_ = 2.53; *p* = 0.0149, respectively), and decreased the number of REM sleep bouts (*t*_(10)_ = 4.99; *p* = 0.0003) (Fig. 2*B*). Furthermore, the duration of NREM sleep bouts was significantly reduced during the 6 h recording period following CNO injection (*t*_(10)_ = 2.56; *p* = 0.0141); there were no changes in wakefulness bout duration (*t*_(10)_ = 0.65; *p* = 0.2656) (Fig. 2*C*). Because of the reduced number of mice that had REM sleep after CNO administration and the scarcity of REM sleep bouts in this group, the treatment effect on REM sleep bout duration was not statistically analyzed (VEH vs CNO: 13.98 ± 0.86 vs 10.64 ± 2.75; Fig. 2*C*).

Figure 2*D* illustrates hour-by-hour changes in the time spent in wakefulness, NREM sleep, and REM sleep (expressed as the percent of total time) as a function of treatment condition during the 6 h after injection. Two-way, repeated-measures ANOVA indicated a significant time effect and treatment condition × time interaction for wakefulness (*F*_(5,50)_ = 15.36; *p* < 0.0001 and *F*_(5,50)_ = 4.98; *p* = 0.0009) and NREM sleep (*F*_(5,50)_ = 12.18; *p* < 0.0001 and *F*_(5,50)_ = 5.49; *p* = 0.0004). CNO administration caused a significant increase in the time in wakefulness during the first hour after injection (*p* = 0.0014) and a reduction of NREM sleep duration during hours 1 (*p* < 0.0001) and 2 (*p* = 0.0188) after injection. For REM sleep, ANOVA revealed a significant time (*F*_(5,50)_ = 5.35; *p* = 0.0005) and drug (*F*_(1,10)_ = 45.12; *p* < 0.0001) effect as well as a treatment × time interaction (*F*_(5,50)_ = 2.69; *p* = 0.0316).

Activation of preoptic glutamatergic neurons caused a significant decrease in the time spent in REM sleep between hours 2-6 after CNO injection (*p* = 0.002, *p* < 0.0001, *p* < 0.0001, *p* < 0.0001, and *p* < 0.0001). Figure 2*E* shows that, relative to control, CNO altered the number of wakefulness, NREM sleep, and REM sleep bouts. ANOVA showed a significant drug and time effect on the number of wake (*F*_(1,10)_ = 6.82; *p* = 0.0260 and *F*_(5,50)_ = 5.32; *p* = 0.0005) and NREM sleep bouts (*F*_(1,10)_ = 6.29; *p* = 0.0311 and *F*_(5,50)_ = 6.77; *p* < 0.0001), and no significant effect on treatment condition × time interaction (*F*_(5,50)_ = 1.41; *p* = 0.2380 and *F*_(5,50)_ = 1.62; *p* = 0.1725). The hour-by-hour *post hoc* analysis revealed that CNO significantly increased the number of wake (*p* = 0.0103, *p* = 0.0036, *p* = 0.0013, and 0.0048) and NREM sleep (*p* = 0.0129, *p* = 0.0033, *p* = 0.0013, and *p* = 0.0033) bouts during hours 2, 3, 4, and 6 after injection. Additionally, there was a significant effect of CNO administration on the number of REM sleep bouts (*F*_(1,10)_ = 26.43; *p* = 0.0004), a significant time effect (*F*_(5,50)_ = 7.26; *p* < 0.0001), and a significant treatment × time interaction (*F*_(5,50)_ = 2.55; *p* = 0.0391). Congruent with the decrease in REM sleep time, the mean number of REM sleep bouts was significantly reduced between hours 2-6 after CNO administration (*p* < 0.0001, *p* < 0.0001, *p* < 0.0001, *p* < 0.0001, and *p* < 0.0001). Figure 2*F* plots the mean bout duration for wakefulness, NREM sleep, and REM sleep. Two-way, repeated-measures ANOVA indicated a significant time effect on the bout duration for wakefulness (*F*_(5,50)_ = 3.74; *p* = 0.0059) and a significant drug effect on bout duration for NREM sleep (*F*_(1,10)_ = 8.14; *p* = 0.0172). *Post hoc* Bonferroni showed that CNO significantly increased the duration of wake bouts (*p* = 0.0067) during the first hour after injection, which accounts for the increase in the time in wakefulness shown in Figure 2*A*. Of note, the large deviation in the mean episode duration is related to 1 mouse that remained awake for the entire hour after injection; removal of this mouse's VEH and CNO data points did not affect statistical significance. Bonferroni tests revealed that NREM sleep episode duration was significantly decreased during hours 1 (*p* = 0.0010) and 2 (*p* = 0.0006) after CNO injection. There were no significant changes in REM sleep episode duration. Figure 3 illustrates the sleep-wake architecture in each Vglut2-Cre mouse (*n* = 11) expressing excitatory hM3Dq receptors in glutamatergic neurons of the medial-lateral preoptic region, after injection of VEH or CNO.

### Activation of medial-lateral preoptic glutamatergic neurons increased NREM and REM sleep latency

**Figure 4.**
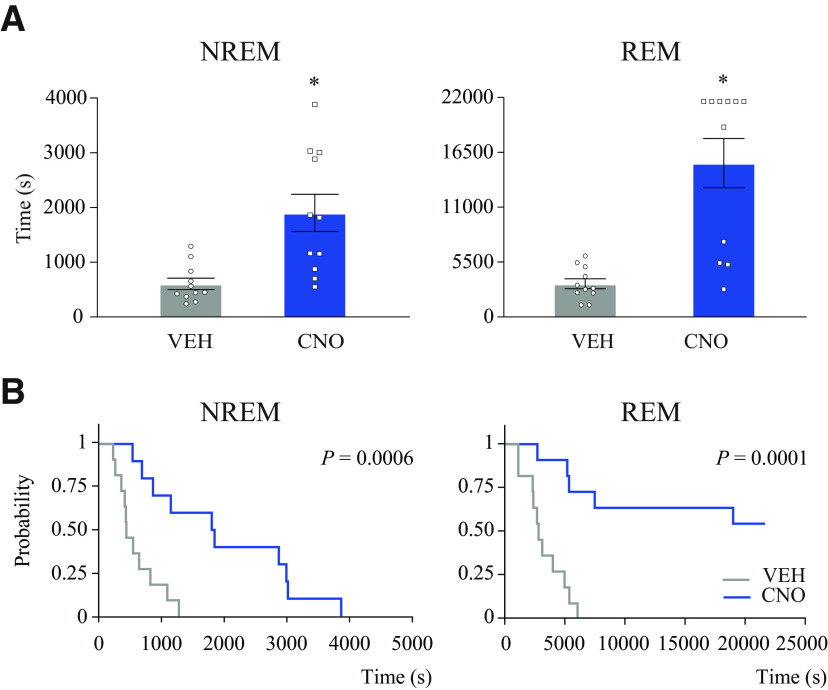
Activation of glutamatergic neurons in the medial-lateral preoptic region increased the latency to NREM and REM sleep. ***A***, Effect of CNO (1.0 mg/kg) injection on NREM (left) and REM sleep (right) latencies in *n* = 11 mice. Data are mean ± SEM. One-tailed paired *t* test was used for statistical comparisons. *Significant difference (*p* < 0.05) relative to control. ***B***, Graphs represent the probability of no NREM sleep (left) and REM sleep (right) generation after injection of VEH or CNO. Survival analysis demonstrated that the probability of no REM sleep occurring remained increased throughout the 6 h recording period and was significantly different between treatment condition in both NREM (*p* = 0.0006) and REM sleep (*p* = 0.0001).

Figure 4 illustrates sleep latencies measured after VEH and CNO injection. The Figure 4*A* graphs plot NREM and REM sleep latencies averaged across the 6 h recording period for all mice. Animals that did not have REM sleep after CNO administration (*n* = 6) were assigned the maximum possible time (total recording time = 21,600 s) as the latency to REM sleep. Relative to VEH, CNO injection significantly increased the latency to both NREM (mean ± SEM = 604.55 ± 102.51 vs 1796.36 ± 378.39, *t*_(10)_ = 2.88; *p* = 0.0081) and REM sleep (3334.55 ± 494.74 vs 15 419.55 ± 2463.17, *t*_(10)_ = 4.76; *p* = 0.0004). Because several mice did not have REM sleep after CNO injection, changes in the latency to the first REM sleep bout (also the latency to NREM sleep onset) were assessed by survival analysis (Vanini and Baghdoyan, 2013). The Figure 4*B* graphs plot the probability of not having NREM sleep and REM sleep after CNO administration. Activation of preoptic glutamatergic neurons significantly increased the probability of not having NREM sleep (*p* = 0.0006) and REM sleep (*p* = 0.0001). Furthermore, relative to control, the probability of REM sleep not occurring after CNO administration remained elevated (>50%) until the end of the recording period.

### Activation of medial-lateral preoptic glutamatergic neurons increased NREM to wake transitions and caused a robust NREM sleep fragmentation

**Figure 5.**
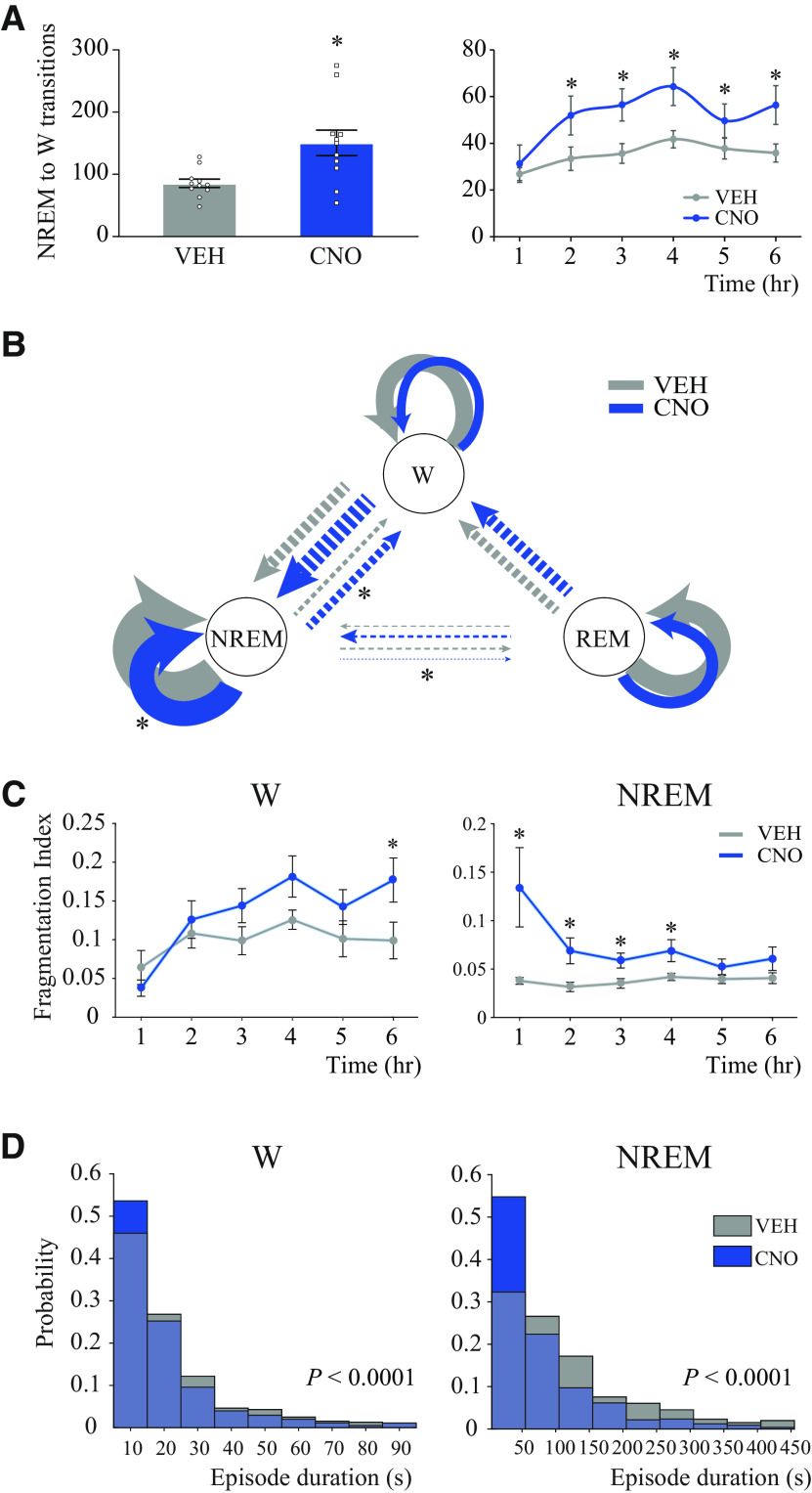
Activation of glutamatergic neurons in the medial-lateral preoptic region increased NREM to wake transitions and caused NREM sleep fragmentation. ***A***, Number of transitions from NREM to wakefulness (W) averaged across the 6 h recording period (left) and per 1 h block (right) after injection of VEH or CNO in *n* = 11 mice. Data are mean ± SEM. One-tailed paired *t* test (6 h block) and two-way repeated-measures ANOVA followed by a Sidak test to correct for multiple comparisons (1 h block analysis) was used for statistical comparisons between treatment conditions. *Significant difference (*p* < 0.05) relative to control. ***B***, Diagram of the Markov model for wakefulness (W)–NREM sleep–REM sleep dynamics after VEH and CNO (1.0 mg/kg) injection in *n* = 11 mice. Circular arrows indicate the probability of remaining within the same state. Straight arrows indicate the probability of transitioning from one state to another. The thickness of the arrows is proportional to the corresponding probability. Two different scales were used: one for the circular arrows and another one for straight arrows; that is, circular arrows were designed with continuous lines, whereas straight arrows were designed with dotted lines. Differences between VEH and CNO were analyzed by means of Wilcoxon matched-pairs rank tests. *Significant difference (*p* < 0.05) relative to control. ***C***, FI calculated from *n* = 11 mice for each 1 h block during wakefulness and NREM sleep after VEH or CNO injection. We defined FI as FI = 1 – p(X/X), being p(X/X) the probability of transitioning from the State X to the same State X. This implies that FI = 1 only if the state is completely fragmented, that is, if the probability of transitioning to the same state equals 0. Error bars indicate SEM. Differences between VEH and CNO were analyzed by means of Wilcoxon matched-pairs rank tests. *p* values were corrected by the Benjamini-Hochberg for a false discovery rate of 5%. *Significant difference (*p* < 0.05) relative to control. ***D***, Histograms represent the probability distribution of episode durations calculated from *n* = 11 mice in units of 10 s of wakefulness and in units of 50 s of NREM sleep. VEH and CNO histograms were overlapped to better appreciate the differences. The difference in the distribution of episode duration between VEH and CNO injection was analyzed by means of a Kolmogorov-Smirnov test. Episode duration after CNO and VEH had a different distribution in both wakefulness (*p* < 0.0001) and NREM sleep (*p* < 0.0001), with increased short and reduced long bouts after CNO administration.

Because activation of preoptic glutamatergic neurons increased the number of wake and NREM sleep bouts, we compared the number of transitions from NREM sleep to wakefulness as a function of treatment condition and time. Figure 5*A* (left) plots the mean number of NREM to wake transitions averaged across the 6 h recording period for all mice. CNO injection significantly increased the number of transitions by 76% (*t*_(10)_ = 3.36; *p* = 0.0036). Figure 5*A* (right) illustrates hour-by-hour changes in NREM to wake transitions during 6 h after VEH and drug administration. ANOVA revealed a significant effect of treatment (*F*_(1,10)_ = 11.35; *p* = 0.0071). There was no treatment × time interaction (*F*_(5,50)_ = 1.96; *p* = 0.1012). Multiple-comparisons *post hoc* tests showed that the number of transitions was significantly increased between postinjection hours 2-6 (*p* = 0.0014, *p* = 0.0001, *p* < 0.0001, *p* = 0.0168, and *p* = 0.0002). There was no significant correlation between REM sleep time and the number of NREM to wake transitions (*r* = −0.4808; *p* = 0.1364).

Based on (1) the increase in the number of NREM sleep bouts, (2) the reduction of NREM sleep bout duration, and (3) the increment in the number of NREM to wake transitions after CNO injection, we hypothesized that the activation of preoptic glutamatergic neurons causes NREM sleep instability. We tested this hypothesis using three different approaches. First, we calculated the state transition probability after VEH and CNO administration by means of a Markov model. Figure 5*B* depicts the probability of transitioning between/within states of wakefulness, NREM sleep, and REM sleep. Consistent with a previous study (Perez-Atencio et al., 2018), the probability of remaining in one state was much higher than the probability of transitioning into a different state. Relative to VEH, activation of glutamatergic neurons in the medial-lateral preoptic region significantly reduced the probability of remaining in NREM sleep (mean ± SEM = 0.9384 ± 0.009 vs 0.9621 ± 0.003, *W* [probability vector value] = −7; *p* = 0.0186), while there was no significant change in the probability of remaining in wakefulness (0.8983 ± 0.0491 vs 0.9205 ± 0.0069, *W* = −24; *p* = 0.1602) or REM sleep (0.9052 ± 0.040 vs 0.9621 ± 0.003, *W* = −7; *p* = 0.2188). Consistent with this evidence, there was an increased probability of transitioning from NREM sleep to wakefulness (0.060 ± 0.010 vs 0.031 ± 0.003, *W* = 64; *p* = 0.0010), while the probability of transitioning from wakefulness to NREM sleep was not affected (0.1017 ± 0.015 vs 0.079 ± 0.007, *W* = 24.0; *p* = 0.1602). Furthermore, as expected because of the drastic reduction in the amount of REM sleep, CNO significantly decreased the probability of transitioning from NREM to REM sleep (0.0017 ± 0.001 vs 0.0072 ± 0.001, *W* = −60; *p* = 0.0024). The probability of entering either wakefulness or NREM sleep from REM sleep was not affected by the activation of the glutamatergic neurons of the medial-lateral preoptic region (0.0833 ± 0.032 vs 0.0625 ± 0.008, *W* = 7; *p* = 0.2188 and 0.011 ± 0.009 vs 0.003 ± 0.001, *W* = 2; *p* = 0.3750, respectively). None of the mice entered REM sleep from wakefulness after VEH, as expected, or CNO administration. Second, to evaluate the stability of sleep-wake states in each 1 h block, we calculated an FI; FI = 1 indicates that the state is maximally unstable and fragmented. Because of the scarcity of REM sleep after CNO administration, we only performed this analysis for wakefulness and NREM sleep states. Figure 5*C* shows that, compared with VEH, stimulation of glutamatergic neurons in the medial-lateral preoptic region significantly fragmented NREM sleep during the first 4 h of the recording period (*W* = 45, *p* = 0.0294; *W* = 54, *p* = 0.0294; *W* = 48, *p* = 0.0315; *W* = 46, *p* = 0.0315). There was no significant correlation between REM sleep time and FI NREM values (*r* = −0.3569; *p* = 0.2785). Additionally, wakefulness was only fragmented during the last hour of the 6-h-long recording period (*W* = 64, *p* = 0.0060). Third, to understand better the effect of the activation of preoptic glutamatergic neurons on sleep and wake episode duration, we compared the distribution of NREM sleep and wake bout duration after VEH and CNO injection. Figure 5*D* shows that the activation of glutamatergic neurons of the medial-lateral preoptic region significantly altered the probability distribution of bout duration by increasing the number of short bouts and decreasing the number of long bouts during both NREM sleep (*p* < 0.0001) and wakefulness (*p* < 0.0001).

### Activation of medial-lateral preoptic glutamatergic neurons reduces body temperature

**Figure 6.**
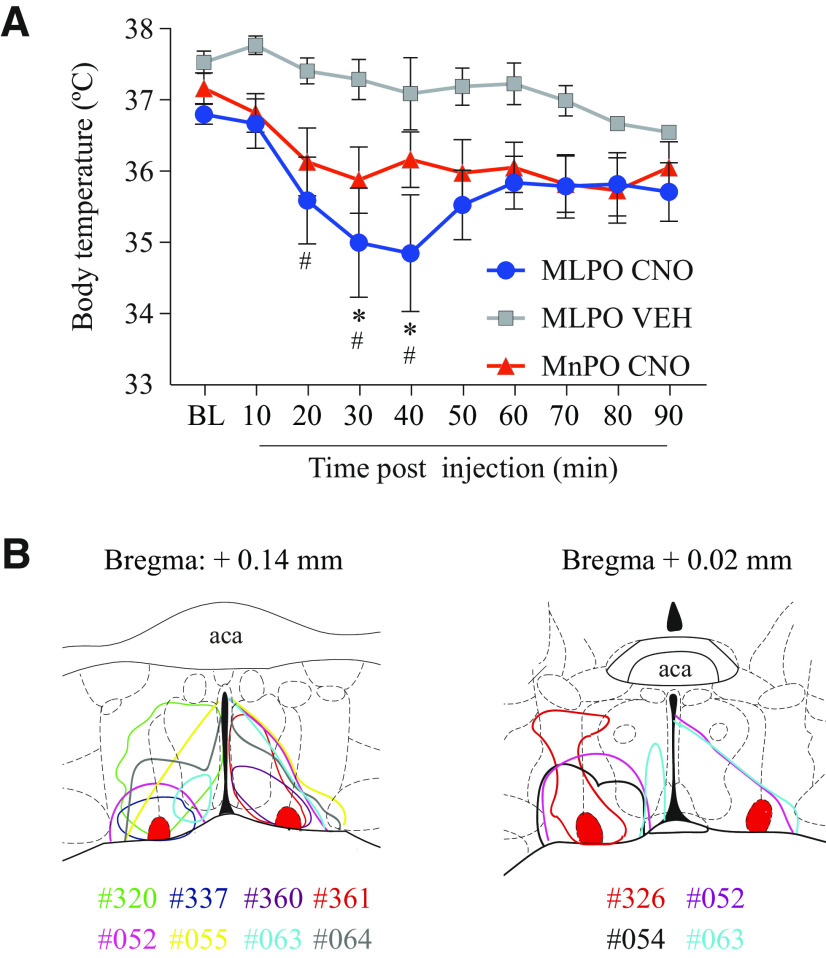
Activation of glutamatergic neurons in the medial-lateral preoptic region causes hypothermia in awake mice. ***A***, The time course of core body temperature in awake mice before and after injection of VEH control solution (*n* = 5 mice) or CNO (1.0 mg/kg) for activation of glutamatergic neurons within the MnPO (*n* = 9) and medial-lateral preoptic region (MLPO; *n* = 10). Temperature values for the MnPO are from Vanini et al. (2020, their Fig. 3; see also their Fig. S4 showing the distribution of the excitatory designer receptor hM3Dq within the MnPO of Vglut2-Cre mice used in that study). Data are mean ± SEM. Two-way ANOVA followed by a *post hoc* Dunnett's test corrected for multiple-comparisons was used for statistical comparisons of mean temperature levels after VEH and CNO injection relative to baseline (BL). Differences in temperature levels between the VEH and the medial-lateral preoptic group, and medial-lateral preoptic versus MnPO glutamatergic group were assessed by Tukey's and Sidak's *post hoc* tests. *Significant difference (*p* < 0.05) in the medial-lateral preoptic group relative to baseline. ^#^Significant difference relative to VEH. ***B***, Color-coded area of hM3Dq receptor expression within the medial-lateral preoptic region of Vglut2-Cre mice used for temperature experiments, represented on coronal schematics of the preoptic area modified from a mouse brain atlas (Paxinos and Franklin, 2001).

Activation of MnPO glutamatergic (Abbott and Saper, 2017; Vanini et al., 2020) and VLPO galaninergic neurons (Kroeger et al., 2018) has been shown in mice to cause hypothermia, which can increase wakefulness and reduce NREM and REM sleep duration (Parmeggiani, 1987). Thus, the effect of glutamatergic neurons in the medial-lateral preoptic region on core body temperature was evaluated before and after CNO injection (*n* = 10 mice), compared with a VEH control group (*n* = 5), and relative to previously published data obtained after activation of glutamatergic neurons in the MnPO (*n* = 9) (Fig. 6). MnPO temperature values used here are from the data reported previously (Vanini et al., 2020). Relative to baseline, a two-way ANOVA revealed a significant time (*F*_(9,210)_ = 2.17; *p* = 0.0252) and treatment (*F*_(2,210)_ = 19.03; *p* < 0.0001) effect. A Dunnett's test demonstrated that, relative to baseline, the activation of glutamatergic neurons in the medial-lateral preoptic region significantly reduced body temperature at 30 and 40 min after injection (*p* = 0.0238 and *p* = 0.0104). Compared with VEH, CNO injection caused hypothermia between 20 and 40 min (Tukey's test; *p* = 0.0340, *p* = 0.0051, and *p* = 0.0064). Relative to the MnPO, there was no significant difference in temperature at any time point. Furthermore, the mean temperature decrease after CNO injection (from min 20-90) was 1.28 ± 0.48°C (mean ± SEM) in the medial-lateral preoptic group and 1.19 ± 0.26°C in the MnPO group, and there was no significant difference in the magnitude of temperature change between both groups (*t*_(0.1738)_ = 13.71; *p* = 0.8645). In a separate experiment, we investigated the time course of mean temperature changes induced by the activation of glutamatergic neurons in the medial-lateral preoptic region (*n* = 5), with respect to the duration of the sleep studies described above. Temperature measurements were obtained before and after CNO injection, every 30 min for 6 h. A one-way, repeated-measures ANOVA showed a significant treatment effect (*F*_(2.428,9.710)_ = 20.08; *p* = 0.0003). *Post hoc* Dunnett's test demonstrated that there was a sustained, significant reduction in body temperature between minute 30 and 360 after CNO administration (*p* = 0.0049, *p* = 0.0235, *p* = 0.0085, *p* = 0.0046, *p* = 0.0131, *p* = 0.0012, *p* = 0.0027, *p* = 0.0003, *p* = 0.0005, *p* = 0.0022, *p* = 0.0030, and *p* = 0.0017).

### CNO administration to control mice without designer receptors did not alter sleep-wake states

**Figure 7.**
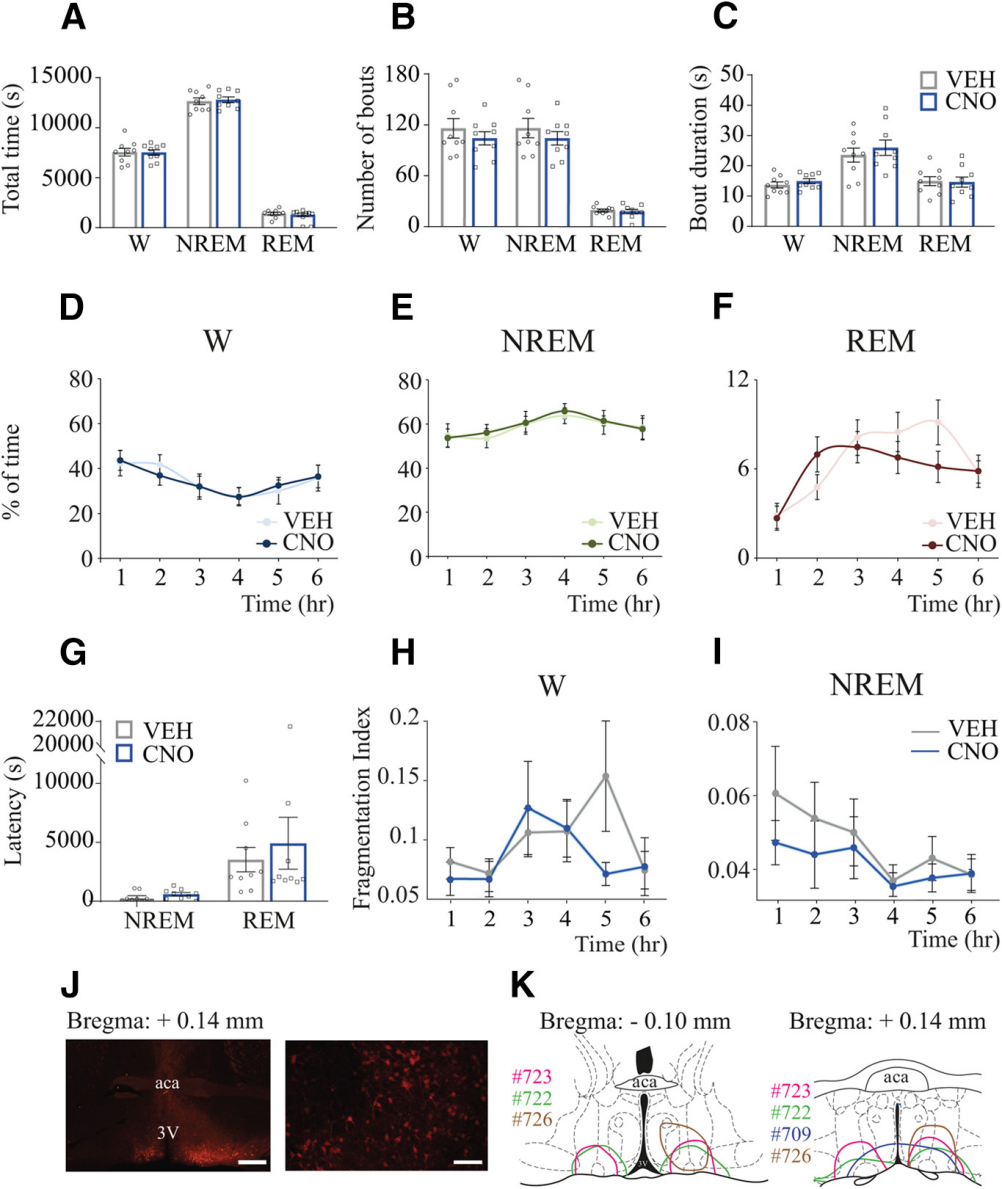
CNO administration to Vglut2-Cre mice that did not express designer receptors did not alter sleep-wake states. Sleep data from Vglut2-Cre mice injected with (1) AAV-hSyn-DIO-hM3D(Gq)-mCherry into the preoptic area but did not express designer receptors (*n* = 5) and (2) the control vector AAV-hSyn-DIO-mCherry (*n* = 4) were pooled together and used as negative controls. ***A***, Group data summarizing total time in wakefulness, NREM sleep, and REM sleep after VEH or CNO (1.0 mg/kg) injection. ***B***, Number of bouts of wakefulness, NREM sleep, and REM sleep. ***C***, Comparison of mean episode duration per state. A one-tailed paired *t* test with Bonferroni correction was used for statistical comparisons between treatment conditions. ***D–F***. Graphs plot the mean time (expressed as percent of total recording time) in wakefulness, NREM sleep, and REM sleep, respectively, for each 1 h block after VEH and CNO administration. Two-way repeated-measures ANOVA followed by a Bonferroni test was used for statistical comparisons. ***G***, Latency to NREM and REM sleep. A one-tailed paired *t* test was used for statistical comparisons between treatment conditions. ***H***, ***I***, FI calculated for NREM and REM sleep, respectively, plotted for each 1 h block after VEH and CNO injection. Wilcoxon matched-pairs rank tests were used for statistical comparisons between treatment conditions. Data are mean ± SEM. ***J***, Left, Right, Low- and high-magnification photographs, respectively, of mCherry immunohistochemical staining (red) corresponding to the fluorescent reporter of the control vector within the VLPO of a Vglut2-Cre mouse. Scale bars: Left, 500 µm; Right, 100 µm. ***K***, Color-coded depiction of control vector injection area, represented on coronal schematics of the preoptic area modified from a mouse brain atlas (Paxinos and Franklin, 2001). Color-matched identification numbers for each mouse used in this study are listed on the left side of each panel. aca, Anterior commissure; 3V, third ventricle.

To rule out any nonspecific effect of CNO or its active metabolites (Ilg et al., 2018), as well as from the cell damage caused by the injection procedure or vector-associated toxicity (Rezai Amin et al., 2019), we included two negative control groups. The effect of 1.0 mg/kg CNO on sleep-wake states was evaluated in a group of mice that received the vector injection into the medial-lateral preoptic region but did not express hM3Dq receptors (*n* = 5), and in a second group injected with the “empty” control vector that only contained the fluorescent reporter mCherry (*n* = 4). Consistent with previous work (Vanini et al., 2020), CNO injection did not alter sleep-wake states in either group. Therefore, the data from all 9 mice were pooled for statistical analysis. The data corresponding to each control group (6 h average) are shown in Table 1. Figure 7*A–C* summarizes group data averaged across the 6 h recording period and shows that CNO injection did not modify the time in wakefulness (*t*_(8)_ = 0.069; *p* = 0.9460), NREM sleep (*t*_(8)_ = 0.351; *p* = 0.7344), or REM sleep (*t*_(8)_ = 0.506; *p* = 0.6265). Neither the number of bouts nor their duration was altered during wakefulness (*t*_(8)_ = 1.182; *p* = 0.2710 and *t*_(8)_ = 1.850; *p* = 0.1014), NREM sleep (*t*_(8)_ = 1.209; *p* = 0.2613 and *t*_(8)_ = 1.215; *p* = 0.2589), or REM sleep (*t*_(8)_ = 0.525; *p* = 0.6137 and *t*_(8)_ = 0.142; *p* = 0.8909). Furthermore, there were no significant changes in the total time (expressed as the percent of total time) in wakefulness, NREM sleep, and REM sleep in the hour-by-hour analysis (Fig. 7*D–F*). Two-way, repeated-measures ANOVA showed no significant effect of the treatment, or treatment × time interaction in wakefulness (*F*_(1,8)_ = 0.005; *p* = 0.9460 and *F*_(5,40)_ = 0.125; *p* = 0.9860), NREM sleep (*F*_(1,8)_ = 0.124; *p* = 0.7344 and *F*_(5,40)_ = 0.054; *p* = 0.9980), and REM sleep (*F*_(1,8)_ = 0.257; *p* = 0.6260 and *F*_(5,40)_ = 2.17; *p* = 0.0767). The latency to NREM or REM sleep was not significantly altered by CNO, as shown in Figure 7*G* (mean time in s ± SEM = 336.1 ± 143.0 vs 598.9 ± 142.0, *t*_(8)_ = 1.986; *p* = 0.0823 and 3530 ± 1034 vs 4929 ± 2202, *t*_(8)_ = 0.662; *p* = 0.5268, respectively). Importantly, neither wakefulness nor NREM sleep was fragmented during the 6 h period after CNO injection (0.075 ± 0.005 vs 0.068 ± 0.003, *W* = −27; *p* = 0.1289; and 0.046 ± 0.005 vs 0.040 ± 0.003, *W* = −21; *p* = 0.2500). Accordingly, no fragmentation was found in any of the six 1 h block analyses for wakefulness (*W* = −27, *p* = 0.1289; *W* = −3.0, *p* = 0.9102; *W* = −11, *p* = 0.5703; *W* = 1.0, *p* > 0.9999; *W* = −19, *p* = 0.3008; *W* = 1.0, *p* > 0.9999) or NREM sleep (*W* = −15, *p* = 0.4258; *W* = −27, *p* = 0.1289; *W* = −11, *p* = 0.5703; *W* = 9.0, *p* = 0.6523; *W* = −13, *p* = 0.4961; *W* = 7.0, *p* = 0.7344) as shown in Figure 7*H*, *I*.

**Table 1.** Sleep-wake parameters obtained from individual, negative control groups as a function of treatment condition*^a^*

	AAV-mCherry (“empty” control vector)		AAV without hM3Dq expression	
	VEH	CNO	VEH	CNO
Time spent in wake (s)	7919 ± 633.5	7730 ± 353.3	7250 ± 484.3	7347 ± 435.4
Time spent in NREM sleep (s)	12443 ± 516.9	12321 ± 347.5	12803 ± 482.7	13166 ± 374.3
Time spent in REM sleep (s)	1239 ± 312.3	1549 ± 77.55	1547 ± 55.6	1087 ± 262.3
No. of wake bouts	126.8 ± 17.54	109.3 ± 5.22	107.2 ± 15.68	100.0 ± 13.88
No. of NREM sleep bouts	126.8 ± 17.72	108.8 ± 5.28	107.8 ± 15.62	100.6 ± 14.09
No. of REM sleep bouts	17.50 ± 2.46	19.75 ± 3.42	20.6 ± 2.22	16.40 ± 3.95
Duration of wake bout (s)	12.84 ± 1.01	14.30 ± 1.22	14.29 ± 1.63	15.36 ± 1.23
Duration of NREM sleep bout (s)	20.93 ± 3.13	22.75 ± 1.04	25.60 ± 3.40	28.56 ± 4.32
Duration of REM sleep bout (s)	14.18 ± 3.17	17.13 ± 2.84	15.52 ± 1.22	12.60 ± 1.70
Latency to NREM sleep (s)	356.3 ± 237.1	278.8 ± 139.6	320 ± 199.2	855 ± 154.7
Latency to REM sleep (s)	4256 ± 1993	1813 ± 125.6	2949 ± 1128	7423 ± 3735

*^a^*Data (*n* = 9 Vglut2-Cre mice) are mean ± SEM. Relative to control (VEH), there were no significant differences. CNO (hM3Dq receptor agonist; 1.0 mg/kg).

### Activation of glutamatergic neurons in the MnPO did not substantially alter sleep-wake states

**Figure 8.**
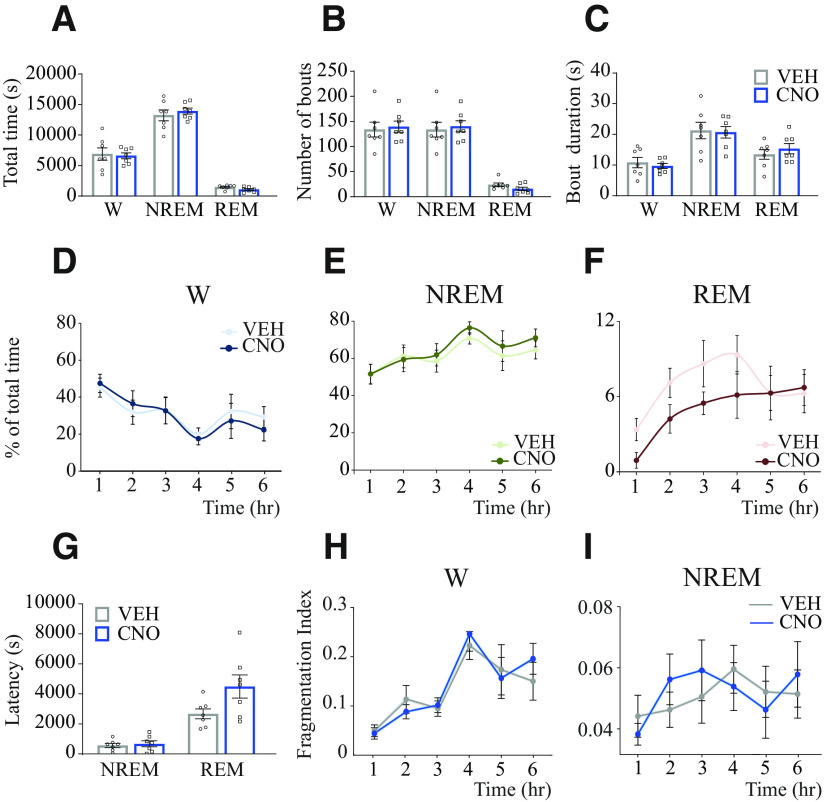
Activation of glutamatergic neurons in the MnPO of the hypothalamus did not substantially alter sleep-wake states. Sleep data from a previous study (collected using identical procedures and experiment design) (Vanini et al., 2020) were reanalyzed and used in the current study as a site-control group. All Vglut2-Cre mice (*n* = 7) included in this control group had confirmed hM3Dq receptor expression in MnPO glutamatergic neurons (for information on the distribution of the excitatory designer receptor hM3Dq within the MnPO, see Vanini et al., 2020, their Fig. S4). ***A***, Group data summarizing total time in wakefulness, NREM sleep, and REM sleep after VEH or CNO (1.0 mg/kg) injection. ***B***, Number of bouts of wakefulness, NREM sleep, and REM sleep. ***C***, Comparison of mean episode duration per state. A one-tailed paired *t* test with Bonferroni correction was used for statistical comparisons between treatment conditions. ***D–F***, Graphs represent the mean time (expressed as percent of total recording time) in wakefulness, NREM sleep, and REM sleep, respectively, for each 1 h block after VEH and CNO administration. Two-way repeated-measures ANOVA followed by a Bonferroni test was used for statistical comparisons. ***G***, Latency to NREM and REM sleep. A one-tailed paired *t* test was used for statistical comparisons. ***H***, ***I***, FI calculated for NREM and REM sleep, respectively, plotted for each 1 h block after VEH and CNO injection. Wilcoxon matched-pairs rank tests were used for statistical comparisons between treatment conditions. Data are mean ± SEM.

To test whether the effects of the activation of glutamatergic neurons in the medial-lateral preoptic region were site-specific, we reanalyzed sleep-wake data from a previous study in which we used the same chemogenetic methods and experiment design to stimulate MnPO glutamatergic neurons in Vglut2-Cre mice (Vanini et al., 2020). Figure 8*A–C* shows that activation of glutamatergic neurons within the MnPO (*n* = 7 mice) did not modify the time in wakefulness (*t*_(6)_ = 0.253; *p* = 0.8087), NREM sleep (*t*_(6)_ = 0.748; *p* = 0.2412), and REM sleep (*t*_(6)_ = 1.738; *p* = 0.066). CNO administration did not alter the number of bouts or bout duration of wakefulness (*t*_(6)_ = 0.577; *p* = 0.2925 and *t*_(6)_ = 0.630; *p* = 0.5519), NREM (*t*_(6)_ = 0.635; *p* = 0.2745 and *t*_(6)_ = 0.223; *p* = 0.8312), and REM sleep (*t*_(6)_ = 2.294; *p* = 0.0616 and *t*_(6)_ = 0.889; *p* = 0.4082). Additionally, there were no significant changes in the time (expressed as the percent of total time) spent in wake, NREM sleep, and REM sleep in the 1 h block analysis (Fig. 8*D–F*). Two-way, repeated-measures ANOVA revealed no significant effect of treatment or treatment × time interaction for wakefulness (*F*_(1,6)_ = 0.06; *p* = 0.809 and *F*_(5,30)_ = 0.37; *p* = 0.866), NREM sleep (*F*_(1,6)_ = 0.16; *p* = 0.7059 and *F*_(5,30)_ = 0.41; *p* = 0.8386), and REM sleep (*F*_(1,6)_ = 3.02; *p* = 0.1328 and *F*_(5,30)_ = 0.91; *p* = 0.4860). Figure 8*G* shows that activation of glutamatergic neurons in the MnPO did not alter the latency to NREM (mean time in s ± SEM = 568.6 ± 136.5 vs 680.0 ± 199.0, *t*_(6)_ = 0.619; *p* = 0.558) or REM sleep (2676 ± 330.3 vs 4496 ± 777.0, *t*_(6)_ = 2.14; *p* = 0.076). Furthermore, activation of MnPO glutamatergic neurons did not cause fragmentation of wake or NREM sleep states (Fig. 8*H*,*I*).

### Activation of medial-lateral preoptic glutamatergic neurons altered EEG features during wakefulness and NREM sleep

**Figure 9.**
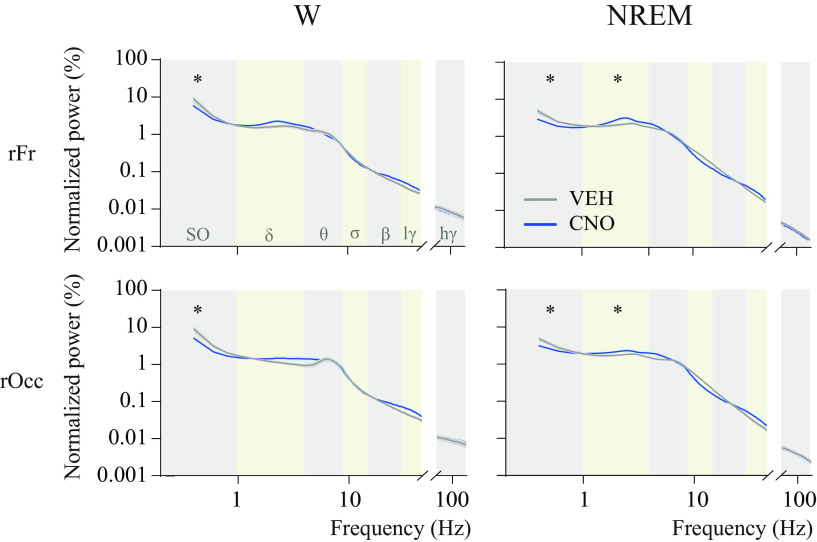
Activation of glutamatergic neurons in the medial-lateral preoptic region decreased the spectral power of slow oscillations and increased δ power. Graphs plot normalized spectral power in the right frontal (rFr) and occipital (rOcc) regions during wakefulness (W) and NREM sleep, after injection of VEH or CNO (1.0 mg/kg) in *n* = 10 mice. Traces represent mean values (thin, dark lines) ± the SEM (shaded area above and below the mean). Alternating vertical-colored bands in the background of the graphs represent frequency ranges. Because notch filters were applied to some recording pairs (i.e., VEH and CNO recordings from the same mouse), frequencies between 45 and 75 Hz were excluded from the analysis. Two-way repeated-measures ANOVA followed by a Sidak test corrected for multiple comparisons was used for statistical comparison of spectral power in each frequency band. *Significant difference (*p* < 0.05) relative to control. SO, Slow oscillations; lγ, low-γ; hγ, high-γ.

This study aimed to determine whether the activation of preoptic glutamatergic neurons, in addition to altering sleep-wake patterns, alters cortical dynamics (i.e., oscillations, connectivity, and complexity) associated with sleep and wakefulness. We thus evaluated local and network-level cortical activity by means of power spectrum, functional and directed connectivity between frontal and occipital cortices, as well as the complexity of EEG signals. Figure 9 shows the mean spectral power from frontal and occipital cortices during wakefulness and NREM sleep. There was a significant treatment effect (CNO) on the spectral power in the occipital cortex during wakefulness (*F*_(1,9)_ = 18.79; *p* = 0.0019), and in the frontal cortex during NREM sleep (*F*_(1,9)_ = 6.46; *p* = 0.0317). There was a significant drug × EEG frequency band interaction for the occipital cortex during wakefulness (*F*_(6,54)_ = 9.33; *p* < 0.0001) and during NREM sleep (*F*_(6,54)_ = 6.41; *p* < 0.0001). No significant treatment effect or treatment × EEG frequency band interaction was found in the frontal cortex during wakefulness (*F*_(1,9)_ = 3.40; *p* = 0.0985 and *F*_(6,54)_ = 2.19; *p* = 0.0952); there was a significant EEG frequency effect (*F*_(6,54)_ = 166.3; *p* < 0.0001). *Post hoc* (Bonferroni) multiple-comparisons analysis revealed that CNO injection significantly decreased the power of slow oscillations (0.5-1 Hz) during wakefulness (*p* = 0.0077, frontal, and *p* < 0.0001, occipital) and NREM sleep (*p* < 0.0001, frontal, and *p* < 0.0001, occipital), and increased δ power during NREM sleep (*p* = 0.0051, frontal, and *p* = 0.0198, occipital). However, the increase in δ power is mainly driven by only 1 mouse that had a relatively large increase after normalization (128%; corrected *p* value after removal of this outlier is 0.0761, frontal, and 0.2480, occipital; Cohen's *d* = 0.6 and 0.4).

**Figure 10.**
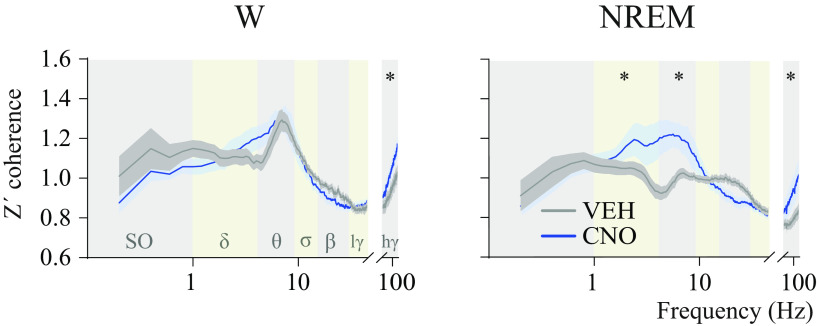
Activation of glutamatergic neurons in the medial-lateral preoptic region increased cortical connectivity. Mean Z'coherence (undirected connectivity) profile between right frontal and occipital as a function of state (wakefulness [W] and NREM sleep) and treatment (VEH and CNO 1.0 mg/kg) in *n* = 10 mice. Traces represent mean values (thin, dark lines) ± the SEM (shaded area above and below the mean). Alternating vertical-colored bands in the background of the graphs represent frequency ranges. Because notch filters were applied to some recording pairs (i.e., VEH and CNO recordings from the same mouse), frequencies between 45 and 75 Hz were excluded from the analysis. *Significant difference (*p* < 0.05) relative to control. SO, Slow oscillations; lγ, low-γ; hγ, high-γ.

Figure 10 plots Z'coherence between frontal and occipital cortices as a function of EEG frequency band and treatment condition. ANOVA revealed a significant treatment × EEG frequency band interaction during wakefulness (*F*_(6,54)_ = 3.34; *p* = 0.0072) and NREM sleep (*F*_(6,54)_ = 6.13; *p* < 0.0001). Furthermore, a *post hoc* multiple-comparisons test demonstrated an increase in high-γ coherence during wakefulness (*p* = 0.0449), as well as δ (*p* = 0.0163), theta (*p* < 0.0001), and high-γ (*p* = 0.0209) Z'coherence during NREM sleep. In accordance with the changes in Z'coherence (undirected functional connectivity), CNO administration altered NSTE (directed connectivity). Mean feedforward and feedback NSTE values for each frequency band during wake and NREM states are shown in Table 2. Compared with VEH, CNO injection significantly altered feedforward connectivity during NREM sleep (*F*_(1,9)_ = 6.60; *p* = 0.0302) but not during wakefulness (*F*_(1,9)_ = 1.86; *p* = 0.2059). Moreover, there was a significant drug × frequency band interaction for the frontal-to-occipital NSTE during NREM (*F*_(5,45)_ = 4.05; *p* = 0.0040) and for the occipital-to-frontal NSTE during wakefulness (*F*_(5,45)_ = 3.71; *p* = 0.0068) and NREM sleep (*F*_(5,45)_ = 4.95; *p* = 0.0011). *Post hoc* multiple-comparisons tests demonstrated that theta frontal-to-occipital connectivity was increased during NREM sleep (*p* = 0.0003). Additionally, theta occipital-to-frontal connectivity was increased during NREM (*p* < 0.0001) and wakefulness (*p* = 0.0052). Compared with VEH injection, CNO did not modify feedforward NSTE during NREM in δ (*p* = 0.0759), σ, β, low-γ, and high-γ (*p* > 0.9999 for each frequency band), as well as the feedback NSTE during wakefulness or NREM in δ (*p* = 0.8974 and *p* > 0.9999, respectively), σ, β, low-γ, and high-γ (*p* > 0.9999 for each frequency band and each behavioral state). To summarize, activation of preoptic glutamatergic neurons enhanced both undirected and directed connectivity in the theta frequency band during NREM sleep as well as undirected functional connectivity in the high-γ band during wakefulness and NREM sleep.

**Table 2.** Directed cortical connectivity during NREM and wake states, by EEG frequency band and as a function of treatment condition*^a^*

EEG frequency band	Occipital-to-frontal (feedback connectivity)				Frontal-to-occipital (feedforward connectivity)			
	Wakefulness		NREM sleep		Wakefulness		NREM sleep	
	VEH	CNO	VEH	CNO	VEH	CNO	VEH	CNO
δ (0.5-4 Hz)	0.033 ± 0.002	0.029 ± 0.002	0.028 ± 0.001	0.031 ± 0.003	0.037 ± 0.002	0.048 ± 0.007	0.052 ± 0.008	0.038 ± 0.002
Theta (4-9 Hz)	0.049 ± 0.003	0.059 ± 0.004	0.035 ± 0.001	0.051 ± 0.004*	0.086 ± 0.006	0.090 ± 0.009*	0.073 ± 0.010	0.050 ± 0.003*
Sigma (9-15 Hz)	0.049 ± 0.002	0.047 ± 0.005	0.049 ± 0.003	0.047 ± 0.005	0.049 ± 0.003	0.050 ± 0.007	0.040 ± 0.004	0.039 ± 0.001
Beta (15-30 Hz)	0.026 ± 0.024	0.023 ± 0.002	0.027 ± 0.001	0.023 ± 0.001	0.0250 ± 0.001	0.022 ± 0.001	0.021 ± 0.001	0.027 ± 0.001
Low γ (30-45 Hz)	0.021 ± 0.001	0.024 ± 0.002	0.022 ± 0.001	0.023 ± 0.001	0.018 ± 0.001	0.019 ± 0.001	0.019 ± 0.001	0.019 ± 0.001
High γ (75-115 Hz)	0.017 ± 0.001	0.020 ± 0.001	0.011 ± 0.001	0.015 ± 0.002	0.020 ± 0.001	0.025 ± 0.001	0.019 ± 0.002	0.014 ± 0.001

*^a^*Data (*n* = 10 Vglut2-Cre mice) are mean ± SEM. A two-way repeated-measures ANOVA followed by Sidak multiple-comparisons test was used for statistical comparisons. CNO (agonist at hM3Dq receptors; 1.0 mg/kg).

*Significant differences relative to control (*p* < 0.05).

**Figure 11.**
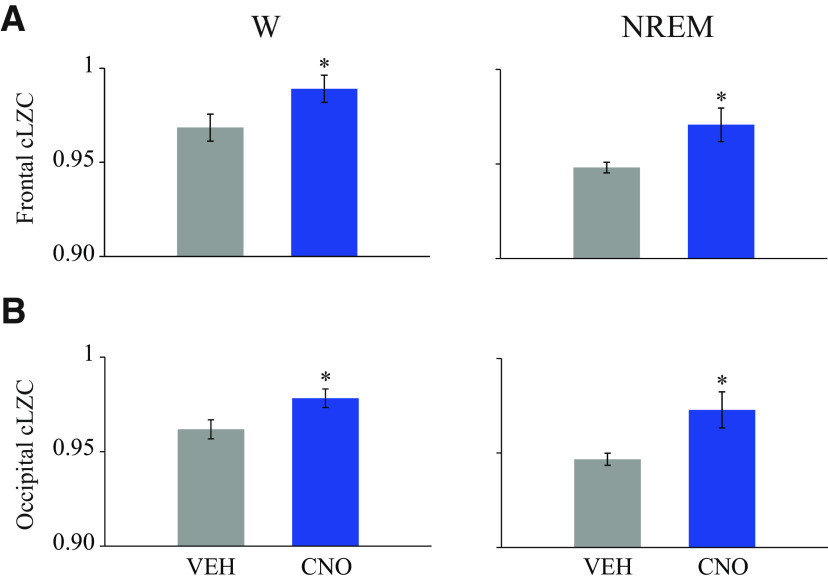
Activation of glutamatergic neurons in the medial-lateral preoptic region increased EEG signal complexity. Graphs plot corrected LZc (cLZc) values during wakefulness (W) and NREM sleep for frontal (***A***) and occipital (***B***) regions. Data are mean ± SEM. Differences between VEH and CNO (1.0 mg/kg) in *n* = 10 mice were analyzed by two-tailed paired *t* tests. *Significant difference (*p* < 0.05) relative to control.

Changes in temporal complexity of the signals evaluated by a corrected LZc analysis are summarized in Figure 11. Relative to VEH injection, CNO increased EEG signal complexity during wakefulness in both frontal (mean ± SEM = 0.9782 ± 0.005 vs 0.9618 ± 0.005; *t*_(9)_ = 2.47, *p* = 0.0354) and occipital regions (0.9891 ± 0.007 vs 0.968 ± 0.007; *t*_(9)_ = 3.72, *p* = 0.0048). Additionally, CNO increased the EEG complexity during NREM sleep in frontal (0.9728 ± 0.009 vs 0.947 ± 0.003; *t*_(9)_ = 3.29, *p* = 0.0094) and occipital regions (0.9708 ± 0.009 vs 0.948 ± 0.003; *t*_(9)_ = 3.13, *p* = 0.0122). Interestingly, EEG complexity levels during NREM sleep after CNO were not significantly different from during wakefulness in the control treatment condition (frontal cortex: *t*_(9)_ = 0.94; *p* = 0.3719 and occipital cortex: *t*_(9)_ = 0.26 *p* = 0.8029).

## Discussion

This study demonstrates that chemogenetic activation of preoptic glutamatergic neurons within the medial-lateral preoptic region of the hypothalamus increases wakefulness, decreases NREM sleep, causes NREM sleep fragmentation and state instability, reduces body temperature, and suppresses REM sleep. Furthermore, these neurons influence cortical oscillations, connectivity, and complexity. The effect on state transitions and cortical dynamics are both novel aspects of this study and significantly advance our understanding on the role of these neurons in arousal state control. A dual role for preoptic neurons in sleep-wake regulation was recently predicted by modeling simulations based on EEG dynamics across the sleep-wake cycle of rats with VLPO neuronal lesions (Lombardi et al., 2020). Together with our previous work (Vanini et al., 2020), and evidence demonstrating that optogenetic stimulation of VLPO glutamatergic neurons projecting to the tuberomammillary nucleus increases wakefulness (Chung et al., 2017), this is among the first studies to empirically identify a subset of preoptic neurons that promotes wakefulness. Importantly, activation of glutamatergic neurons in the MnPO (control site) had no substantial effect on sleep-wake duration, sleep latency, and consolidation. The nonsignificant trend toward an increase in the latency and decrease in the duration of REM sleep during the first 4 h after CNO injection is congruent with previous work from our group showing that (in an analysis of only 3 h after CNO administration) MnPO glutamatergic neurons only reduced REM sleep quantity (Vanini et al., 2020). Together, these data suggest that glutamatergic neurons in the MnPO destabilize REM sleep and support the conclusion that the effect of the activation of glutamatergic neurons in the medial-lateral preoptic region on sleep-wake states is site-specific.

The preoptic area contributes to REM sleep regulation. Cell-specific excitotoxic lesions in a region medial and dorsal to the VLPO (termed “extended” VLPO) decreases REM sleep (Lu et al., 2002). Subsets of preoptic neurons are maximally active during spontaneous REM sleep bouts, during periods of sleep restriction when REM sleep pressure is the highest, and during REM sleep rebound following REM sleep deprivation (Lu et al., 2002; Suntsova et al., 2002; Gvilia et al., 2006; Takahashi et al., 2009; Sakai, 2011; M. A. Alam et al., 2014). Furthermore, activation of preoptic GABAergic neurons that innervate the tuberomammillary nucleus increases REM sleep (Chung et al., 2017). Here we show that activation of glutamatergic neurons in the medial-lateral preoptic region decreased REM sleep time. Indeed, this state was eliminated for 6 h in 55% of the mice. To the best of our knowledge, this is the first direct demonstration that preoptic glutamatergic neurons can, directly or indirectly, suppress REM sleep generation. The remarkable reduction in REM sleep may result from several factors. First, the profound sleep fragmentation induced by activation of preoptic glutamatergic neurons can reduce REM sleep because its propensity builds up during prior NREM sleep bouts and spontaneous REM sleep generation requires NREM sleep occurrence (Benington and Heller, 1994; Le Bon, 2020). This is particularly evident in 2 mice that had a substantial increase in NREM sleep (45% in #559 and 19% in #541), but their sleep was highly fragmented and REM sleep was virtually eliminated. However, while NREM sleep fragmentation had a duration of 4 h, REM sleep quantity was reduced or totally suppressed for 6 h after CNO administration; and there was no negative correlation between REM sleep time and sleep fragmentation or the number of NREM-wake transitions. Furthermore, there was no REM homeostatic response after sleep fragmentation, as both number and duration of REM sleep bouts remained unchanged. Another potential mechanism is by a direct or indirect activation of brain circuits that inhibit REM sleep generation. The preoptic region is reciprocally connected with wake-promoting monoaminergic systems and hypocretin/orexin-containing neurons in the perifornical region of the hypothalamus (Zardetto-Smith and Johnson, 1995; Sherin et al., 1998; Chou et al., 2002; Uschakov et al., 2006). Furthermore, the ventrolateral periaqueductal gray receives projections from preoptic neurons (Uschakov et al., 2009; Hsieh et al., 2011) and GABAergic neurons within the periaqueductal gray region exert a powerful inhibitory control on REM sleep generation (Sastre et al., 1996; Vanini et al., 2007; Sapin et al., 2009; Weber et al., 2018). Identification of local and distant target neurons of wake-promoting glutamatergic neurons in the medial-lateral preoptic region will be critical for a circuit-based understanding of their role in REM sleep control. This is especially important because the ability of these neurons to promote wakefulness as well as enhance cortical dynamics while suppressing REM sleep might serve the function of preventing REM-like intrusions after the initiation of waking consciousness. Last, preoptic neurons play an important role in thermoregulation (McGinty et al., 2001). Activation of MnPO glutamatergic (Abbott and Saper, 2017; Vanini et al., 2020) and VLPO galaninergic neurons (Kroeger et al., 2018) induces significant hypothermia in mice, and exposure to cold temperature increases wakefulness and reduces REM sleep in rodents (Roussel et al., 1984; Amici et al., 1998; Baracchi et al., 2008; Sato et al., 2015). Glutamatergic neurons that cause hypothermia (some of which also promote sleep) are mainly localized within the MnPO and medial preoptic area (Zhang et al., 2015; Abbott and Saper, 2017; Harding et al., 2018; Vanini et al., 2020). Here we demonstrate that activation of glutamatergic neurons in the medial-lateral preoptic region causes hypothermia and that the reduction of body temperature was sustained during the 6 h recording period. Interestingly, the severity of the hypothermia induced by activation of glutamatergic neurons in the MnPO and medial-lateral preoptic region was comparable (i.e., there was no significant difference), and CNO administration to MnPO mice did not cause sleep fragmentation or REM suppression. These results suggest that hypothermia and sleep fragmentation caused by activation of glutamatergic neurons in the medial-lateral preoptic region may not be dissociated and may causally contribute to the prevention of REM sleep generation.

This study shows that activation of glutamatergic neurons in the medial-lateral preoptic region causes a transient increase in wakefulness and a reduction in NREM sleep. Additionally, there was a robust, long-lasting NREM sleep fragmentation and state instability resembling the disrupted sleep pattern observed in sleep apnea (Guilleminault et al., 1976; Kimoff, 1996), Alzheimer's disease (Lim et al., 2014), and aging (Lim et al., 2014; J. Li et al., 2018). Similarly, chemogenetic stimulation of cholinergic neurons in the basal forebrain, lateral to the preoptic area, increases wakefulness and fragments sleep (Anaclet et al., 2015); and optical stimulation of basal forebrain parvalbumin neurons induces brief, rapid arousals from sleep (McKenna et al., 2020). Relative to basal forebrain glutamatergic neurons, optogenetic stimulation increases wakefulness (Xu et al., 2015), whereas activation of these neurons using chemogenetic strategies did not significantly alter sleep-wake states (Anaclet et al., 2015). Importantly, none of the mice in this study expressed excitatory designer receptors in the basal forebrain region, confirming again the specificity of the effects of our stimulation region. Given the frequent and brief arousals from sleep observed after CNO administration, we speculate that glutamatergic neurons in the medial-lateral preoptic region may initiate, but not maintain, wakefulness. Furthermore, these neurons may be part of a brain network that produces rapid arousals from sleep in response to endogenous or environmental alert signals.

Given the considerable diversity of preoptic neurons, genetically targeting glutamatergic/Vglut2 neurons for optogenetic or chemogenetic studies of this region does not necessarily restrict the manipulation to a single functional cell group. For example, Moffitt et al. (2018) reported that many excitatory neurons (Vglut2^+^) expressed GABA synthetic genes, and a few Vglut2^+^ neurons in the preoptic area also express the vesicular GABA transporter. Based on this evidence, we cannot rule out that more than one neuronal type was stimulated in the present work, which may explain the substantive increase in NREM sleep time observed in 2 of the mice contrasting with the rest of the cohort in the sleep study. Thus, it is becoming evident that further studies will require a more nuanced approach, identifying and manipulating these neurons based on specific projection sites or neurochemical markers (Chung et al., 2017; Harding et al., 2018; Reitz et al., 2020).

Glutamatergic neurons in the medial-lateral preoptic altered several sleep and wake EEG features related to cortical dynamics. Specifically, CNO administration increased (1) theta and γ coherence, (2) cortical connectivity, and (3) EEG complexity during NREM sleep. These are electrophysiologic traits of more activated states. Indeed, EEG theta and γ coherence, directed connectivity, and complexity are typically highest during wakefulness (Abasolo et al., 2015; Pal et al., 2016, 2020; Brito et al., 2020; Mondino et al., 2020) and progressively decline as sleep deepens (Abasolo et al., 2015; Pal et al., 2016; Bandt, 2017; Gonzalez et al., 2019, 2020; Migliorelli et al., 2019). Together, these EEG changes and the reduction of the spectral power of slow oscillations after CNO injection provide evidence for a “lighter” NREM sleep state. Increased EEG complexity and high-γ coherence are, respectively, suggestive of a higher number of brain network interactions (Guevara Erra et al., 2016) and more alertness during wakefulness (Castro et al., 2013). In the current study, we indeed found that CNO administration increased high-γ coherence, EEG complexity, and feedback connectivity, while reducing the power of slow oscillations during wakefulness. Therefore, these data support the interpretation that activation of glutamatergic neurons in the medial-lateral preoptic region produces an activated EEG state with features that correlate with enhanced wakefulness. Sleep consolidation (Ward et al., 2009) and sleep quality (i.e., slow wave activity and slow oscillations during NREM sleep) (Bellesi et al., 2014; Schreiner et al., 2018; J. Kim et al., 2019; Hahn et al., 2020) as well as REM sleep (Boyce et al., 2017) are crucial for normal cognition. Thus, because of the substantial disruption of sleep patterns and cortical dynamics produced during NREM sleep by the activation of preoptic glutamatergic neurons, further studies are needed to examine the impact on attention and memory processes.

Collectively, our results support the interpretation that glutamatergic neurons in the medial-lateral preoptic region may initiate, but not maintain, wakefulness from sleep, and their inactivation may be necessary for NREM stability and REM sleep initiation. The present work is limited by including only male mice and stimulation strategies as well as by the lack of data on the effects of these preoptic neurons on sleep-wake states during the dark phase. Additionally, future studies are needed to identify relevant projection pathways and neuronal targets mediating their wakefulness-promoting effects. Furthermore, these findings encourage future circuit-based studies to examine the relevance of these neurons in the generation of wakefulness in response to endogenous or environmental alert signals during sleep, while suppressing REM-like intrusions. These data also have translational implications as they potentially inform the etiology of debilitating, disrupted sleep patterns observed in aging, sleep apnea, and dementia. Last, this study advances sleep neurobiology by providing empirical evidence supporting the hypothesis that the preoptic area is not exclusively somnogenic but rather plays a dual role in the regulation of both sleep and wakefulness.
